# Synthesis of Novel Arsonolipids and Development of Novel Arsonoliposome Types

**DOI:** 10.3390/pharmaceutics14081649

**Published:** 2022-08-08

**Authors:** Spyridon Mourtas, Konstantina Papadia, Golfo G. Kordopati, Panayiotis V. Ioannou, Sophia G. Antimisiaris, Gerasimos M. Tsivgoulis

**Affiliations:** 1 Laboratory of Pharmaceutical Technology, Department of Pharmacy, School of Health Sciences, University of Patras, 26510 Rio Patras, Greece; 2 Department of Chemistry, University of Patras, 26510 Rio Patras, Greece; 3 Institute of Chemical Engineering Sciences, Foundation for Research and Technology Hellas (FORTH/ICES), 26504 Rio Patras, Greece

**Keywords:** 2-hydroethylarsonic acid, arsono lipids, arsono cholesterol, ether-based liposomes

## Abstract

Arsonolipids represent a class of arsenic-containing compounds with interesting biological properties either as monomers or as nanostructure forming components, such as arsonoliposomes that possess selective anticancer activity as proven by in vitro and in vivo studies. In this work, we describe, for the first time, the synthesis of novel arsono-containing lipids where the alkyl groups are connected through stable ether bonds. It is expected that this class of arsonolipids, compared with the corresponding ester linked, will have higher chemical stability. To accomplish this task, a new methodology of general application was developed, where a small arsono compound, 2-hydroxyethylarsonic acid, when protected with thiophenol, can be used in an efficient and simple way as a building block for the synthesis of arsono-containing lipids as well as other arsono-containing biomolecules. Thus, besides the above-mentioned arsonolipid, an arsono cholesterol derivative was also obtained. Both ether arsonolipid and arsono cholesterol were able to form liposomes having similar physicochemical properties and integrity to conventional arsonoliposomes. Furthermore, a preliminary in vitro anticancer potential assessment of the novel ether arsonolipid containing liposomes against human prostate cancer (PC-3) and Lewis lung carcinoma (LLC) cells showed significant activity (dose- and time-dependent), which was similar to that of the conventional arsonoliposomes (studied before). Given the fact that novel arsonolipids may be more stable compared to the ones used in conventional arsonoliposomes, the current results justify further exploitation of the novel compounds by in vitro and in vivo studies.

## 1. Introduction

Although exposure to arsenic (As) has been related to severe adverse health effects [1,2,3,4,5,6,7,8], arsenic trioxide (As_2_O_3_) has been used as a drug for the treatment of various diseases, while it has also shown remarkable therapeutic efficacy in patients with acute promyelocytic leukemia (APL) [9,10]. In fact, a series of clinical trials with As_2_O_3_ has confirmed its benefit in the therapy of APL, and it was formally approved as a drug by the State Drug Administration (SDA) and the Food and Drug Administration (FDA) [11], while its role in the treatment of other malignancies remains to be determined [12]. Recently, it has been reported that some organic arsenic species have a strong anticancer effect against solid tumors [13]. Among them, arsenical C-glucoside derivatives revealed promising antitumor activity [14], while the organic arsenic-containing compounds Darinaparsin (DAR) and 4-(N-(S-glutathionylacetyl) amino) phenylarsonous acid (GSAO) are in clinical trials of the U.S. Food and Drug Administration for the treatment of cancers such as leukemia, lymphomas and solid tumors [13,15,16].

**Figure 1:**
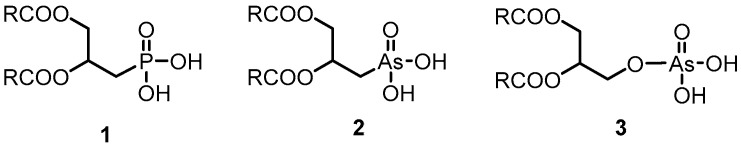
General structure of phosphonolipids **1**, arsonolipids **2** and O-As glycerol lipid analogs **3**.

Arsonolipids **2** (Figure 1) represent another class of arsenic-containing organic compounds, initially synthesized as analogs of phosphonolipids **1**. In fact, the two structures are identical, with the exception of the atom of phosphorus, which is replaced by arsenic (Figure 1) [17,18]. The arsonolipid derivatives of structure **2**, containing a C-As bond, were synthesized in an effort to prepare arsenic-containing lipid derivatives of phospholipids, overcoming the hydrolytic lability of the RO-As bond in the analogs of type **3** [19].

Indeed, arsonolipids **2,** and especially liposomes containing them, that from hereon will be mentioned as conventional arsonoliposomes, have shown remarkable stability and interesting biophysical [20,21,22,23,24], biochemical [25,26], and biological properties [27,28,29,30,31]. Thus, arsonolipids **2** were demonstrated to form liposomes, rods, and cubes, depending on the fatty acyl chain length [20], be efficient transporters of divalent cations [21], inhibit enzymes by interacting with metal cations in their active sites [21], and be poor substrates for phospholipase A2 [26]. Furthermore, conventional asronoliposomes have shown anti-leishmanial and trypanocidal activities [29]. Moreover, conventional arsonoliposomes have shown selective toxicity toward certain cancer cells [27,28,31], demonstrating high toxicity against specific cancer cells while being substantially less toxic towards normal cells, as proven by in vitro [27,28,32] and in vivo studies [28,33]. Recently, the loading of doxorubicin (DOX) into conventional arsonoliposomes revealed possible interesting synergistic effects [34], and folic acid (FA) conjugated and DOX-loaded arsonoliposomes were investigated as targeted therapeutics for the treatment of triple-negative breast cancer (TNBC) [35]. 

Other types of As-lipids, such as the arsonium-containing lipophosphoramides, have been used in poly-functional nano-carriers with antibacterial activity and eukaryotic cell transfection [36].

The mechanism proposed for the anticancer activity of conventional arsonoliposomes was linked with the reduction in the pentavalent arsenic to its more toxic trivalent form (As(V) to As(III), e.g., from AsO(OH)_2_ to As(SR)_2_). This reduction is enhanced in areas where increased concentrations of thiols prevail, which is the case for some types of cancer cells [37]. For this reason, the synthesis of novel lipids containing the As(V) group is of high interest, and several synthetic methods leading to different arsenic-containing organic compounds have been proposed [38,39,40,41].

**Figure 2:**
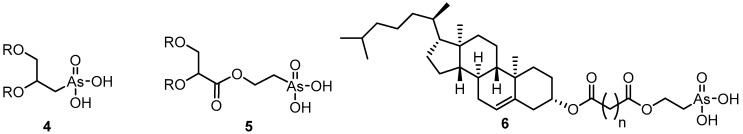
Structures of the targeted arsono-containing lipids.

In this paper, the synthesis of a new class of arsonolipids containing saturated lipids connected through ether bonds (instead of ester bonds that are usually found in phospholipids) using a new methodology of general application is described. It is expected that this class of lipids will have higher stability over hydrolysis and oxidation based on analogous studies in the case of phospholipids [42,43,44]. Initially, the synthesis of the ether containing arsonolipids of type **4** (Figure 2) was attempted using *rac*-2,3-dihydroxypropylarsonic acid, a key building block that has already been used in the synthesis of the ester arsonolipids of type **2 [17,45]**. As it was found, this approach did not give the targeted compounds, probably reflecting the relative instability of the As-C bond. To overcome this difficulty, a different approach was followed where the etherified glycerol lipidic part was synthesized separately and attached to the arsenic polar head via an ester bond (molecules of type **5**). In this approach, 2-hydroxytheylarsonic acid was used as a key building block.

To examine the applicability of this method for the synthesis of other arsono lipid compounds, this synthetic approach was performed for the synthesis of an arsono cholesterol derivative (As-Chol) of type **6** (Figure 2) where the cholesterol moiety is connected to the arsono head group. Steroids have an excellent ability to penetrate cell membranes and bind to the nuclear and membrane receptors [46]. More specifically, cholesterol derivatives are widely studied for their anticancer properties, while several anticancer agents have been chemically conjugated with cholesterol molecules to enhance their pharmacokinetic behavior, cellular uptake, target specificity, and safety [47,48]. Therefore, novel methods in the synthesis of arseno-containing cholesterol molecules of type **6**, such as those described here, can be of interest.

To the best of our knowledge, this is the first time that 2-hydroxyethylarsonic acid has been used as a key molecule for the synthesis of arseno-containing lipids. Furthermore, for the first time, As-lipid molecules of type **5** and an As-Chol derivative of type **6** are synthesized.

The aim of the current study is to identify a synthetic approach for the synthesis of novel arsonolipid compounds and to examine if the new arsonolipids could be used for the formation of a new class of arsonoliposomes. It is of special interest to investigate if, indeed, such novel arsonoliposomes would retain their capability to form arsonoliposomes with high integrity, thus having the potential to be used as therapeutic systems, and more importantly, if they would retain their anticancer properties. For this, polyethylene glycol (PEG) coated novel arsonoliposomes were constructed with the same lipid compositions that were previously found to confer high integrity to conventional arsonoliposomes [24]. As control preparations, conventional arsonoliposomes were also constructed, and the integrity of the different classes of arsonoliposomes was evaluated by the calcein leakage study under identical experimental conditions [24]. Furthermore, in order to be sure that the novel arsonolipid containing arsonoliposomes retain their anticancer properties, their cytotoxicity towards two different cancer cell types was evaluated in comparison to conventional arsonoliposomes. 

## 2. Materials and Methods

### 2.1. Materials

All chemicals used for the syntheses were purchased from Sigma-Aldrich OM, Athens, Greece, and were used without further purification, while all required anhydrous solvents were dried with molecular sieves for at least 48 h prior to use. Silica gel 230–400 mesh (for flash column chromatography) and silica gel 60 F254 plates (for thin layer chromatography (TLC)) were obtained from Merck (Darmstadt, Germany). TLC spot detection was carried out by UV light or by charring with an aqueous solution of K_2_CO_3_/KMnO_4_. NMR spectra were recorded on a Brucker DPX 400 MHz instrument (Peoria, IL, USA). MS were recorded on a QTRAP system with an ESI source (Applied Biosystems, Waltham, MA, USA).

For arsonoliposome preparation, 1,2-Distearoyl-sn-glycerol-3-phosphatidyl-choline (DSPC), and 1,2-Distearoyl-sn-glycerol-3-phosphatidylethanolamine-N-(methoxy(polyethylene-glycol)-2000) (PEG2000) were purchased from Lipoid, Ludwigshafen, Germany. Cholesterol (Chol), Fetal Calf Serum (FCS) and Sephadex G50 (course), calcein, and Sepharose CL-4B were purchased from Sigma-Aldrich OM, Athens, Greece. A bath sonicator (Branson, Danbury, CT, USA) and a microtip-probe sonicator (Sonics and Materials, Newtown, CT, USA) were used for liposome preparation. Fluorescence intensity (FI) of samples was measured with a Shimatzu RF-5301PC spectrofluorophotometer (Kyoto, Japan) at 37 ± 0.1 °C. In all cases, 5-nm slits were used. The Cell Viability MTT assay was carried out with 3-(4,5-Dimethylthiazol-2-yl)-2,5-diphenyltetrazolium bromide (MTT) reagent, which was purchased by Sigma-Aldrich. All other reagents and chemicals used were of analytical grade and were purchased from Sigma-Aldrich.

### 2.2. Synthetic Procedures

Octadecyl-1-methanesulfonate (**8**): Το οctadecyl alcohol (**7**) (1 g, 3.70 mmol) dissolved in anhydrous DCM (3.5 mL), *N,N*-diisopropylethylamine (DIPEA) (0.77 mL, 4.44 mmol) was added, and the mixture was cooled in an ice bath at 0 °C. Methanesulfonyl chloride (0.31 mL, 4.07 mmol) diluted in DCM (0.5 mL) was added slowly, and the reaction mixture was stirred at 0 °C for *ca*. 15–30 min. Then, the reaction mixture was transferred to a separatory funnel and extracted with water (5 mL). The organic phase was washed with 10% aq. citric acid, 10% aq. Na_2_CO_3_, 10% aq. citric acid and finally water (5 mL each). The organic layer was dried (Na_2_SO_4_) and concentrated on a rotary evaporator to *ca.* 3–4 mL, and a mixture of Et_2_O/*n*-hexane, 1:1, (10 mL) was added. The resulting solution was kept at 4 °C. The suspension that was formed was filtered, washed with Et_2_O/*n*-hexane, 1:1, (2 × 5 mL) and dried to afford white crystals (**8**) (1.12 g, 83% yield). ^1^H-NMR of **8** is presented in Figure S1. ESI-MS of **8** is also analyzed in the Supplementary Materials File.

*rac*-1-(2-(octadecyloxy) propoxy)octadecane (**10**): Potassium hydroxide (21 mg, 0.375 mmol) was dissolved in DMSO (900 μL), *rac*-propane-1,2-diol (4.57 μL, 0.0624 mmol) was added, and the mixture was stirred at room temperature (rt) for 45 min. Then, octadecyl methanesulfonate (**8**) (50.0 mg, 0.144 mmol) was added, and the reaction mixture was allowed to stir at rt for 5 d. After the reaction completion, the reaction mixture was diluted with water (5 mL) and extracted with Et_2_O (3 × 5 mL). The combined organic layers were washed with water (3 × 5 mL), and the final organic layer was dried (Na_2_SO_4_) and evaporated. The oily residue that was obtained was purified by column chromatography (silica gel, *n*-hexane/Et_2_O, 95:5) to obtain an oily product (**10**) (22.5 mg, 62% yield). ^1^H-NMR of **10** is presented in Figure S2. ESI-MS of **10** is also analyzed in the Supplementary Materials File.

2-hydroxyethylarsonic acid (**15**): Arsenious trioxide (As_2_O_3_) (1.98 g, 10.0 mmol) was placed in a flask,13.33 M aq. NaOH (6 mL, 80.0 mmol) was added, and the mixture was kept under stirring. 2-chloroethanol (**14**) (1.37 mL, 20.4 mmol) was added drop-wise, while the reaction temperature was maintained between 15 and 17 °C. When the addition of 2-chloroethanol was complete, the reaction mixture was stirred for 2 h at 15–17 °C and continued stirring overnight at rt. Then, the reaction mixture was acidified with conc. HCl until pH = 9 and the mixture was freeze-dried. To the resulting solid, absolute EtOH (20 mL) was added, and the solid was filtered and washed with absolute EtOH (2 × 20 mL). The solid was dissolved in water (24 mL) and acidified with aq. HCl until pH = 2.5. The solution was then freeze-dried, and the resulting solid was filtered and washed with absolute EtOH (2 × 16 mL). The ethanol filtrates containing 2-hydroxyethylarsonic acid were collected and evaporated until the volume was about 10 mL and were kept at 2–4 °C. The oily residue that was formed (**15**) was collected and dried (2.71 g, 80% yield). ^1^H-NMR of **15** is presented in Figure S3.

Diphenyl 2-hydroxyethylarsonodithionite (**16**): To a mixture of 2-hydroxyethylarsonic acid (**15**) (30.6 mg; 0.18 mmol) dissolved in CH_3_OH (700 μL), PhSH (73.7 μL; 0.72 mmol) was added, and the mixture was stirred at rt for 2 h. After reaction completion, CH_3_OH was evaporated and Et_2_O (2 mL) was added. To this solution, anhydrous MgSO_4_ was added, and the mixture was vortexed and allowed to stand for 10 min. Solid MgSO_4_ was removed, and solvents were evaporated. The oily residue that was formed was re-suspended in Et_2_O, and the solvent was evaporated again. The final oily residue (**16**) that was obtained was used in the next step without further purification. ^1^H-NMR of **16** is presented in Figure S4.

2-(palmitoyloxy)ethylarsonic acid (**18**): To the oily diphenyl 2-hydroxyethyl arsonodithionite (**16**) (60.9 mg, 0.18 mmol), DMAP (1.10 mg; 0.009 mmol) dissolved in CHCl_3_ (1.6 mL) and pyridine (52.6 μL; 0.65 mmol) were added. The reaction mixture was cooled at 2–4 °C, and then palmitoyl chloride (180.2 μL; 0.594 mmol) was added slowly for 20 min and the reaction mixture was stirred at 2–4 °C for 2 h. CHCl_3_ was evaporated, and the oily residue that was formed was suspended in Et_2_O (3 mL) and water (0.7 mL). Then, 35% aq. H_2_O_2_ (34.8 μL; 0.405 mmol) was added, and the oxidation process was allowed at rt for 4–6 h. The organic layer/solid was separated from the aqueous phase. The organic layer/solid was initially washed with water (2 × 3 mL)) and further concentrated. The solid (**18**) that was formed was finally washed with *n*-hexane and dried (45.0 mg, 61% total yield from **16**). ^1^H-NMR of **18** is presented in Figure S5; ^13^C-NMR of **18** is presented in Figures S6 and S7.

3-O-benzyl-sn-Glycerol/3-(benzyloxy)propane-1,2-diol (**21**): To NaH (0.51 gr as 60% sodium hydride (*w/w*) in mineral oil; 21.2 mmol) dissolved in THF (15 mL), 1,2-*O*-isopropylidene-sn-glycerol (**19**) (2 g, 15.1 mmol) was added and the mixture was stirred at rt for 30 min. To this, benzyl bromide (2.16 mL; 18.2 mmol) was added in three portions for 30 min, and the reaction mixture was stirred overnight at rt. Then, 10% aq. Na_2_CO_3_ (30 mL) was added, and the mixture was stirred at rt for 10 min. Subsequently, the mixture was extracted with Et_2_O (2 × 30 mL), and the combined organic layers were washed with H_2_O (3 × 30 mL) and then concentrated. The oily intermediate product 4-((benzyloxy)methyl)-2,2-dimethyl-1,3-dioxolane that was obtained was directly hydrolyzed with a mixture of DCM/H_2_O/TFA, 10:1:2, (26 mL) at rt for 30 min. In the hydrolysis mixture, 10% aq. Na_2_CO_3_ was added until pH = 10, and this was washed with *n*-hexane (3 × 10 mL). The aqueous phase was further saturated with solid NaCl and extracted with EtOAc (3 × 10 mL). The organic phases were collected, dried (Na_2_SO_4_), and after evaporation, an oily product (**21**) was formed (6.49 gr, 94% yield). ^1^H-NMR of **21** is presented in Figure S8.

*rac*-1-((2,3-bis(octadecyloxy)propoxy)methyl)benzene (**22**) and *rac*-2,3-bis(octadecyloxy) propan-1-ol (**23**): A mixture of 3-benzyl-sn-glycerol (**21**) (213 mg; 1.17 mmol) and NaH (173 mg as 60% sodium hydride (*w/w*) in mineral oil; 4.33 mmol) in DMSO (20 mL) was stirred at 40 °C for 1 h. Then octadecyl methanesulfonate (**8**) (855.7 mg; 2.45 mmol) was added and the reaction mixture was stirred at 40 °C for 72 h. Subsequently, the reaction mixture was transferred to a separatory funnel and extracted with water (30 mL) and EtOAc (20 mL). The organic layer was washed with water (3 × 30 mL), dried (Na_2_SO_4_), and after evaporation, a white waxy solid was formed that was purified by column chromatography (silica gel, *n*-hexane/EtOAc, 50:1), obtaining a white solid (**22**) (401.7 mg, 50% yield). *rac*-1-((2,3-bis(octadecyloxy)propoxy)methyl)benzene (**22**) (401.7 mg, 0.585 mmol) was further subjected to hydrogenolysis in EtOAc (5 mL) in the presence of a small quantity of CH_3_OH) and Palladium 10% *w/w* on Carbon catalyst (Pd-C) overnight. The reaction mixture was filtered, and the filtrate was evaporated obtaining a solid (**23**), which was finally recrystallized by anhydrous EtOH (2 mL) (338.0 mg, 97% yield). ^1^H-NMR of **22** is presented in Figure S9; ^1^H-NMR of **23** is presented in Figure S10; ESI-MS of both **22** and **23** are analyzed in the Supplementary Materials File.

*rac*-2,3-bis(octadecyloxy)propanoic acid (**24**): To a mixture of 2,3-bis(octadecyloxy) propan-1-ol (**23**) (417 mg; 0.698 mmol) in DCM (20 mL) at 25–27 °C, a solution of pyridinium dichromate (PDC) (0.965 mg; 3.492 mmol) in DMF (12.5 mL) was added, and the reaction mixture was stirred at 25–27 °C overnight. Then, DCM was evaporated, and the remaining solution was diluted with Et_2_O (45 mL) and extracted with 1 N HCl (45 mL). The organic phase was washed with 10% Na_2_CO_3_ (30 mL) and H_2_O (20 mL), and the combined aqueous phases were washed with Et_2_O (2 × 20 mL). To the aqueous phase, another portion of Et_2_O (30 mL) was added, and then it was slowly acidified with 12 N HCl to pH = 2. The organic phase was washed with water (3 × 20 mL), dried (Na_2_SO_4_), and finally concentrated to give a white solid. This was recrystallized by anhydrous EtOH to afford a white solid (**24**) (260.6 mg, 61% yield). ^1^H-NMR of **24** is presented in Figure S11; ESI-MS is also analyzed in the Supplementary Materials File.

*rac*-2,3-bis(octadecyloxy)propanoyl chloride (**26**): To a solution of rac-2,3-bis(octadecyloxy)propanoic acid (**24**) (260.6 mg, 0.426 mmol) in toluene (2.85 mL), thionyl chloride (154.7 μL, 2.13 mmol) was added, and the reaction mixture was stirred at 60 °C overnight. Then, the reaction mixture was evaporated (the oily residue was re-dissolved in toluene (5 mL) and evaporated (twice), and the oily product (**25**) that was formed was used directly in the next step.

*rac*-2-(2,3-bis(octadecyloxy)ethylarsonic acid (As-Lipid) (**27**): A freshly prepared diphenyl 2-hydroxyethyl arsonodithionite (**16**) (96.1 mg, 0.284 mmol) and DMAP (0.615 mg; 0.0050 mmol) were dissolved in a mixture of CHCl_3_ (1400 μL) and pyridine (37.8 μL, 0.469 mmol). The mixture was cooled at 2–4 °C, this was added to the oily *rac*-2,3-bis(octadecyloxy)propanoyl chloride (**26**) (268.2 mg, 0.426 mmol) (freshly prepared), and the reaction mixture was stirred at 2–4 °C for 2 h. Then, CHCl_3_ was evaporated, and the remaining oily residue was suspended in Et_2_O (4.7 mL) and water (1.1 mL). 35% aq. H_2_O_2_ (55.0 μL, 0.640 mmol) was added, and the reaction mixture was stirred at rt, overnight. The organic layer/solid was separated from the aqueous phase, and the combined organic layer/solid was initially washed with water (2 × 3 mL)) and further concentrated. The solid that was formed was recrystallized from dioxane (2 mL), washed and dried to obtain a white solid (**27**) (133.1 mg, 61% total yield from **16**). ^1^H-NMR of **27** is presented in Figure S12; ^13^C-NMR of **27** is presented in Figure S13; ^1^H-^13^C HSQC NMR is presented in Figure S14; ^1^H NMR of **24** vs ^1^H NMR of **27** is presented in Figure S15.

Cholesterol-3-O-Succinic Acid monoester (**29**): Cholesterol (**28**) (1.00 g, 2.59 mmol), succinic anhydride (1.29 g, 12.9 mmol) and DMAP (0.316 g, 2.59 mmol) were dissolved in DCM (17 mL) and the reaction mixture was stirred at rt for about 48 h. Then, most of the DCM was evaporated, and to the remaining mixture, acetic acid (15 mL) was added. The precipitate that was formed was filtered and washed with acetic acid and dried to obtain a solid (**29**) (1.01 g, 80% yield). ^1^H-NMR of **29** is presented in Figure S16; ^13^C-NMR of **29** is presented in Figure S17.

As-Chol (**32**): In Cholesterol-3-O-Succinic Acid monoester (**29**) (400 mg, 0.822 mmol) dissolved in toluene (4 mL), thionyl chloride (0.3 mL, 4.14 mmol) was added, and the reaction mixture was stirred at 55 °C overnight. Then, the reaction mixture was evaporated, and the oily residue that was formed was re-dissolved in toluene and evaporated (twice). The oily product (**30**) that was formed was used directly in the next step. A freshly prepared diphenyl 2-hydroxyethyl arsonodithionite (**16**) (186.0 mg, 0.55 mmol) and DMAP (1.18 mg; 0.00968 mmol) were dissolved in a mixture of CHCl_3_ (2.7 mL) and pyridine (72.8 μL, 0.904 mmol). The mixture was cooled at 2–4 °C and then added to the oily chlorinated cholesterol-3-O-succinic acid monoester (**30**) (399.0 mg, 0.82 mmol) (freshly prepared), and the reaction mixture was stirred at 2–4 °C for 2 h. CHCl_3_ was evaporated and the remaining oily residue (**31**) was suspended in Et_2_O (9 mL) and water (2.1 mL). To this suspension, 35% H_2_O_2_ (106 μL, 1.23 mmol) was added and the reaction mixture was stirred overnight at rt. Then, the organic layer/solid was separated from the aqueous phase. The combined organic layer/solid was initially washed with water (2 × 5 mL) and further concentrated. The solid that was formed was further washed with acetone to afford a white solid (**32**) (203.7 mg, 58% total yield from **29**). ^1^H-NMR of **32** is presented in Figure S18; ^13^C-NMR of **32** is presented in Figure S19; ^1^H-NMR of **29** vs ^1^H-NMR of **32** is presented in Figure S20.

### 2.3. NMR Sample Preparation—Operation

Samples of 5–20 mg in about 600–700 μL of the appropriate deuterated solvent were prepared and transferred to the NMR tube directly before analysis. NMR spectra were recorded at 25 °C using 5 mm NMR tubes. Chemical shift assignments, reported in ppm, were referenced to the corresponding deuterated solvent peaks (CDCl_3_, CD_3_OD, DMSO-*d*_6_).

### 2.4. Liposome Preparation Procedures

For the preparation of arsonolipid-containing liposomes, the ‘one step method’ was used [49]. The molecular composition of the different arsonoliposome preparations was: (a) DSPC/Chol/PEG2000/As-ether-lipid (38.7/31.9/5.2/24.2), for the novel As-ether-lipid liposomes, where the attachment of the alkyl chains to the glycerol backbone is realized through ether bonds, (b) DSPC/Chol/PEG2000/As-ester-lipid (38.7/31.9/5.2/24.2), for the conventional arsonolipid containing liposomes, where the attachment of the alkyl chains to the glycerol backbone is realized through ester bonds (As-ester-liposomes), (c) DSPC/Chol/PEG2000/As-Chol (38.7/24.2/7.7/5.24/24.2), for the novel As-Chol liposomes. In brief, lipids (DSPC, Chol, PEG2000, and As-lipid or As-Chol) as powders were mixed in PBS pH 7.40 (or a solution of calcein (100 mM with pH 7.40 and adjusted with NaCl to an osmolarity of 300 mOsm) when the liposomes were intended for integrity studies) and they were magnetically stirred vigorously on a hot plate for 3 h at 75–80 °C. After the formation of liposomes, the samples were left to anneal for at least 1 h at the liposome preparation temperature used in each case. In order to reduce the liposome size, the liposomes were sonicated using a probe sonicator equipped with a tapered micro tip for two 10 min cycles until the initially turbid liposomal suspension was clarified. Following sonication, the liposome suspension was left to stand for 2 h at 60 °C in order to anneal any structural defects. The titanium fragments and any multilamellar vesicles or liposomal aggregates were removed by centrifugation at 10,000× *g* for 15 min. In the case of calcein liposomes, the non-encapsulated material was removed by size exclusion chromatography on a Sepharose 4B-CL (40 × 1 cm) column eluted with PBS, pH 7.40. The exact liposome phospholipid content of the final liposome formulation was measured by the Stewart assay, a colorimetric method that is routinely used for the quantification of phospholipids [50].

### 2.5. Liposome Size Distribution, ζ-Potential, and Stability Studies

The particle size distribution (mean hydrodynamic diameter and polydispersity index) of vesicle dispersions (0.2 mg/mL lipid, in 10 mM PBS, pH 7.40) was measured by dynamic light scattering (DLS) technique (Malvern Nano-ZS; Malvern Instruments, Worcestershire, UK) at 25 °C at a 173-degree angle. Zeta potential (ζ-potential) was measured for the same samples (dispersed in 10 mM PBS, pH 7.40) at 25 °C by the same instrument (utilizing the Doppler electrophoresis technique). The physical stability of some of the vesicle dispersions in buffer was monitored by measuring the mean diameter, polydispersity index (PDI) and ζ-potential at specified time periods during storage at 4 °C for a period of 40 days.

### 2.6. Liposome Integrity Studies

The integrity of As-liposomes and control liposomes was evaluated by incubation of calcein-encapsulating liposomes (1 mg/mL) in PBS (pH 7.40) as well as in FCS (80% *v*/*v*) at 37 °C. Calcein was encapsulated in the liposomes at 100 mM concentration and calcein latency (%) and integrity (%) values were calculated at various time points by taking samples from the incubated liposomes and measuring fluorescence intensity (FI), as previously described [51,52]. In brief, at each time point, 20 ul of vesicle dispersions were taken and diluted in 4 mL of PBS buffer. The FI was measured (EX 470 nm, EM 520 nm) before and after the addition of Triton X-100 at a final concentration of 1% *v/v* (which ensures complete liposome disruption and release of all encapsulated (and latent) dye). Latency (%) was calculated from the equation: Latency (%) = 100 × [(1.1 × Fat) − Fbt]/(1.1 × Fat), where Fbt and Fat are calcein fluorescence intensities before and after the addition of Triton X-100, respectively (for Fat, multiplication by 1.1 was applied for correction due to dilution). For evaluation of the specific disruptive effect that serum proteins cause on liposome membranes, calcein retention (%) of vesicles was calculated from the latency of the liposomes during incubation in buffer, and the corresponding (at the same time point) latency in FCS, according to equation: Retention (%) = 100 × [Latency-in-FCS]/[Latency-in-buffer]. 

### 2.7. Cytotoxicity Study

Two types of cells were used in this study: (i) human prostatic carcinoma cell line (PC-3) and (ii) Lewis lung carcinoma cell line (LLC). Cells were obtained from ATCC (American Type Culture Collection, Manassas, VA, USA) and provided by Prof. G.T. Stathopoulos (Medical School, University of Patras, Patras, Greece). All cells were grown in RPMI 1640 medium supplemented with 10% (Fetal Bovine Serum) FBS and 1% antibiotic-antimycotic solution (Invitrogen, Carlsbad, CA, USA). The cells were cultured at 37 °C, 5% CO_2_/saturated humidity. Medium was changed every 2–3 d. 

The cytotoxicity of the various types of liposomes towards cancer cells was evaluated by the corresponding reduction in cell viability after 24 h (for high As-lipid concentrations) and after 48 and 72 h (for low As-lipid concentrations) by the MTT assay, as previously described [34]. In brief, cells were seeded overnight at 37 °C at a density of 11 × 10^4^ cells per well or 9 × 10^4^ cells per well for the 24 or 48 h studies, respectively, in 96-well plates until almost confluent and then incubated for 24 or 48 h at 37 °C (5% CO_2_/saturated humidity) with 0.1 mL RPMI and 0.1 mL of As-liposomes. All formulations were pre-filtrated through a 0.22 um Millipore filter. The medium/PBS (*v/v*) ratio was kept constant. Cell viability was determined by the MTT method [53]. For this, after the 24 and 48 h incubations with the cells, the medium was removed, and the cells were washed three times with PBS before adding 0.1 mL of fresh medium containing 0.5 mg/mL solution of MTT. Two hours later, 0.1 mL of acidified isopropanol (0.33%) was added to each well in order to disrupt the cells and solubilize the colored formazan crystals that formed. The optical density of the controls and samples was measured at 570 nm (Multiscan EX plate reader, Thermo, Waltham, MA, USA). Viability (%) was calculated by the equation: Viability (%) = (OD_570_ sample − OD_570_ background)/(OD_570_ control − OD_570_ background) × 100, where OD_570_ control corresponds to untreated cells (or PBS control) and OD_570_ background to MTT without cells). 1% Triton X-100 was used as a positive control of cytotoxicity and resulted in viability values < 5% for all cells.

## 3. Results and Discussion

### 3.1. Synthetic Part

**Scheme 1:**
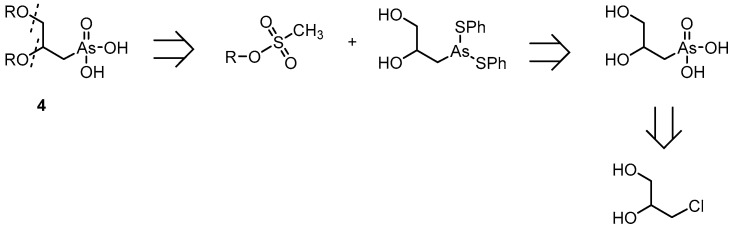
Retrosynthetic analysis for the synthesis of ether arsonolipids **4**.

For the synthesis of the target compound **4** (R = CH_3_-(CH_2_)_17_-), the retrosynthetic approach of Scheme 1 was initially considered, where either *rac*-2,3-dihydroxypropylarsonic acid or its thiophenyl derivative (*rac*-diphenyl 2,3-dihydroxypropylarsonodithionite) and an alkyl methanesulfonate are reacted. This is the most straightforward approach, after the introduction of *rac*-2,3-dihydroxypropylarsonic acid (or its thiophenyl derivative) as building blocks for the synthesis of arsono-containing ester lipid derivatives by Tsivgoulis et al. [17,38,45,54]. In this procedure, a Williamson ether synthesis between alkyl methanesulfonates and the alkoxy ions of *rac*-2,3-dihydroxypropylarsonic acid or its thiophenyl derivative (*rac*-diphenyl 2,3-dihydroxypropylarsonodithionite) would produce the desired products of type **4**.

**Scheme 2:**
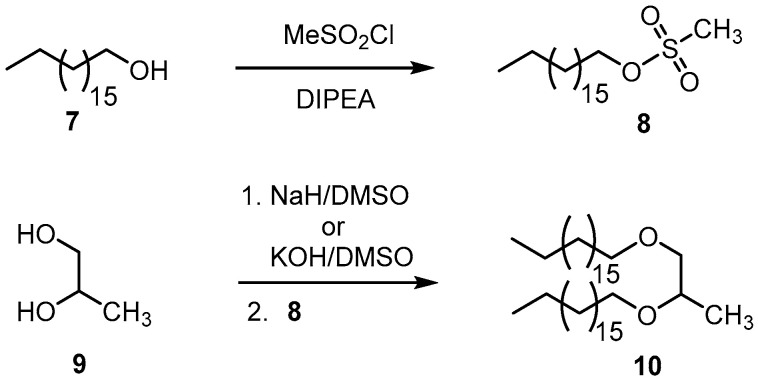
Procedure followed for the synthesis of the ether lipid derivative **10**.

In order to find the mildest conditions for this reaction, *rac*-propane-1,2-diol **9**, which is commercially available and structurally closely related to *rac*-2,3-dihydroxypropylarsonic acid, was used as a model (Scheme 2). As depicted in Scheme 2, octadecyl methanesulfonate **8** was synthesized by mesylation of octadecan-1-ol in the presence of *N,N*-diisopropylethylamine (DIPEA). To enable the ether formation, *rac*-propane-1,2-diol **9** was converted to the corresponding alkoxy dianion with NaH (or deprotonated with KOH) in DMSO and was then reacted with octadecyl methanesulfonate **8** to form the desired ether product **10**.

**Scheme 3:**
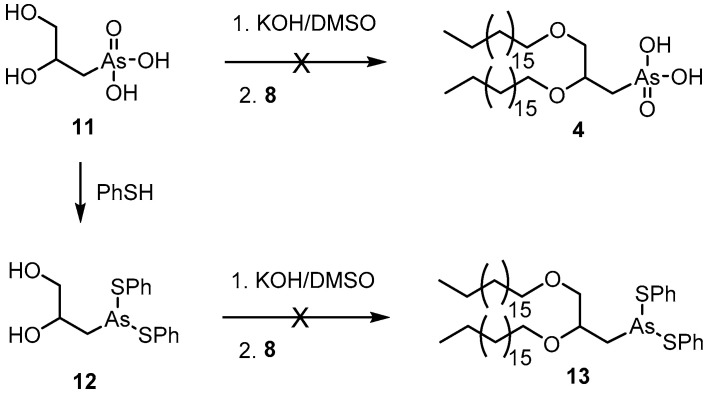
Unsuccessful methods for the synthesis of ether arsonolipids of type **4**.

Based on the reaction conditions found from the experiments with the model diol, the synthesis of ether lipid **4** (Scheme 3) was attempted. Both *rac*-2,3-dihydroxypropylarsonic acid **11** [38] and its thiophenyl derivative **12** [17,45] were used as substrates for the etherification reaction with the octadecyl methanesulfonate **8** under the aforementioned conditions (NaH or KOH in DMSO), either at room temperature or at 60 °C. As it was found, neither **11** nor **12** gave the desired ether lipid **4** or **13**, respectively. Additional attempts to realize the reaction by using mono-potassium and di-potassium salts of *rac*-2,3-dihydroxypropylarsonic acid **11** as starting materials were also unsuccessful.

**Scheme 4:**
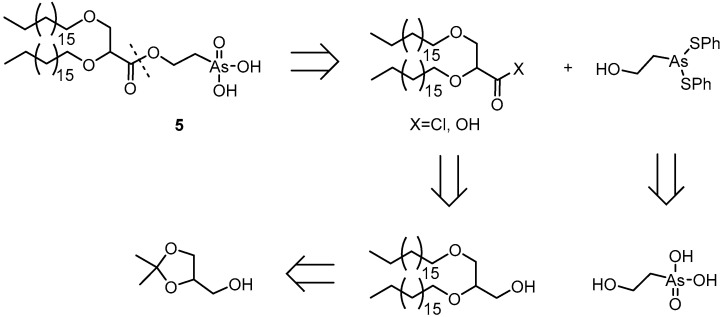
Retrosynthetic analysis for the synthesis of arsenic ether arsonolipids of type **5** (As-ether-lipids)**,** where the attachment of the alkyl chains to the glycerol backbone is realized through ether bonds, while an ester linkage between the lipidic part and 2-hydroxyethylarsonic acid is realized.

An alternative method to obtain arsono lipids with the alkyl groups connected to the glycerol skeleton through ether bonds is presented in the retrosynthetic approach of Scheme 4. In this case, arsono lipids of the general formula **5** can be obtained. The main advantage of this approach is that the Williamson etherification is performed at an earlier step where the arsonic group is not present, thus overcoming the above problems related to the inadequate stability of the C-As bond in the reaction conditions. As depicted in Scheme 4, the insertion of the arsono group is realized through the reaction of 2-hydroxyethylarsonic acid (or diphenyl 2-hydroxyethylarsonodithionite) with a lipid acyl chloride (or a lipid carboxylic acid).

**Scheme 5:**
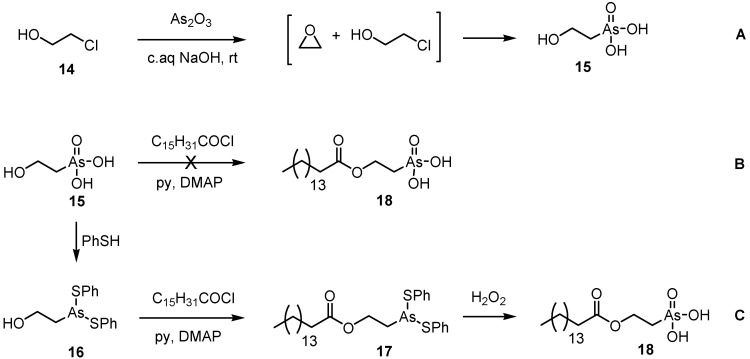
Conditions for: (**A**) the synthesis of: 2-hydroxyarsonic acid **15**; (**B**) the unsuccessful esterification of **15** with palmitoyl chloride and (**C**) the synthesis of 2-(palmitoyloxy)ethylarsonic acid **18** by acylation of diphenyl 2-hydroxyethylarsonodithionite with palmitoyl chloride.

The Meyer reaction was used for the synthesis of 2-hydroxyethylarsonic acid, in which aqueous alkaline arsenite (Na_3_AsO_3_) reacts with alkyl halides or other substrates such as *rac, R* or *S* glycidols to give arsonic acids [55,56]. Thus, 2-chloroethanol **14** reacted with sodium arsenite (As_2_O_3_) in concentrated aq. NaOH (to form Na_3_AsO_3_). The reaction temperature for this reaction was maintained between 15 and 17 °C. The desired product **15** was isolated and purified for further use after appropriate work-up and isolation (Scheme 5A). Since it is known that the As-C bond is not very stable in the presence of acylating agents [57], the esterification reaction of 2-hydroxethylyarsonic acid with acyl chlorides was firstly studied with palmitoyl chloride (as a model). Initially, the esterification was attempted in the presence of pyridine (py) and catalytic amount of 4-(dimethylamino)pyridine DMAP (Scheme 5B), but as it was found, under these conditions, no product was formed. However, when 2-hydroxyethylarsonic acid **15** was protected with thiophenol to form diphenyl 2-hydroxyethylarsonodithionite **16** and subsequently reacted with palmitoyl chloride in the presence of pyridine and a catalytic amount of DMAP followed by oxidation of the produced As-dithiophenyl protected lipid **17** by hydrogen peroxide (Scheme 5C), the desired arsono lipid **18** was obtained.

**Scheme 6:**
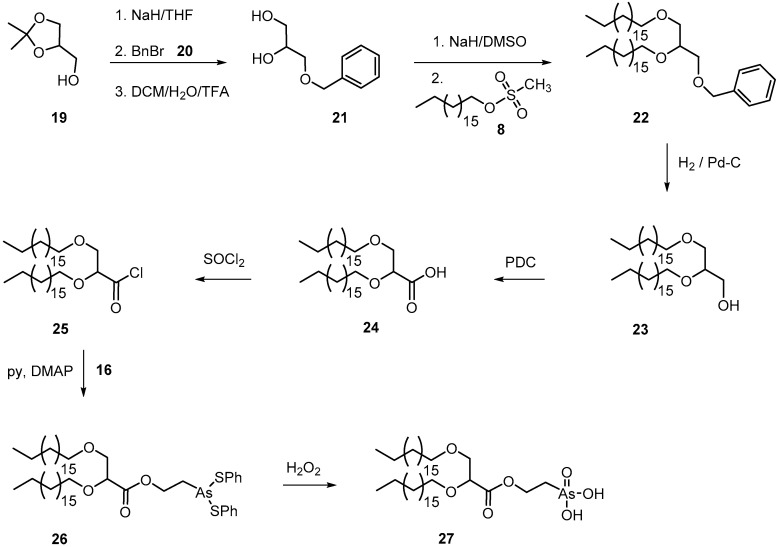
Method for the synthesis of arsono-containing ether lipid of type **5** (As-ether-lipid, **27**).

In the next step, this approach that was successfully applied in the synthesis of the model lipid derivative **18**, was used for the synthesis of the target arsonolipids of type **5**. In this case, the esterification of diphenyl 2-hydroxyethylarsonodithionite **16** requires a lipid acyl chloride of type **25** (Scheme 6). 

The synthesis started with the reaction of the commercially available 1,2-isopropylidene-*rac*-glycerol **19** and benzyl bromide **20** to the corresponding 1-O-Benzyl-*rac*-glycerol **21**, which was further reacted with octadecyl methanesulfonate **8** to produce the lipid ether compound **22** (Scheme 6). This was treated with H_2_/Pd-C, and the derived *rac*-2,3-bis(octadecyloxy)propan-1-ol **23** was oxidized by pyridinium dichromate (PDC) to *rac*-2,3-bis(octadecyloxy)propanoic acid **24,** which was converted to the corresponding acid chloride derivative *rac*-2,3-bis(octadecyloxy)propanoyl chloride **25** using thionyl chloride. In the next step, **25** was reacted with diphenyl 2-hydroxyethylarsonodithionite **16** in the presence of pyridine and catalytic amount of DMAP to the corresponding diphenyl 2-(2,3-bis(octadecyloxy) propanoyloxy)ethyl arsonodithionite **26**. In the final step, **26** was oxidized to the desired lipid arsonic acid **27** (Scheme 6).

**Scheme 7:**
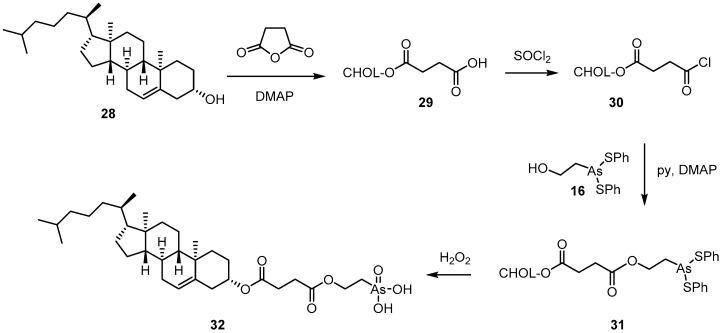
Method for the synthesis of arsono cholesterol derivative of type **6** (As-Chol **32**).

Since the most critical step during the synthesis of arsono lipids of type **5**, following the retrosynthetic approach of Scheme 4, is the introduction of the arsenic group, the importance of 2-hydroxyethylarsonic acid **15** as a common intermediate is apparent. Thus, **15** can be easily converted by its reaction with thiophenol to 2-hydroxyethylarsonodithionite **16,** which, in turn, is a key building block in the synthesis of arsono-containing lipids (or other arsono-containing biomolecules) through an acylation reaction. To explore the generality of this method for the synthesis of arsono-containing biomolecules, **16** was used in the synthesis of other types of arsono-containing lipids with significant biological importance and more complicated structure such as the cholesterol derivative **32** (Scheme 7). Thus, cholesterol **28** was reacted with succinic anhydride, and the produced cholesterol-3-O-succinic acid monoester **29** was converted to the corresponding acyl chloride **30**. Coupling between **30** and **16** successfully gave the intermediate **31,** which was finally oxidized to the target As-Chol derivative **32**.

**Figure 3:**
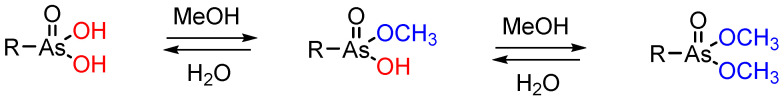
Methylation reactions of the arsonic group when methanol is present.

It should be noticed that when arsonic acids such as **18**, **27** and **32** are mixed with methanol, significant changes to the appearance of their spectra are observed as a result of the fast exchange of the arsonic hydroxyl groups with methoxy ones (Figure 3), and this is in agreement to our previous studies [58].

### 3.2. Comparison of Novel Arsonoliposome with Conventional Arsonoliposomes

The purpose of this part of the current study was to evaluate if the novel lipids can form arsonoliposomes with: (i) nanosize and narrow size distribution, together with high integrity (especially in the presence of serum proteins); properties that ensure their potential for in vivo delivery, and (ii) intrinsic anticancer potential. Since conventional arsonoliposomes have been proven (by numerous in vitro and in vivo studies [27,28,31,32,33,34,35]) to have such properties, a direct comparison of the novel arsonoliposome types with conventional arsonoliposomes was realized herein.

#### 3.2.1. Arsono Liposome Physicochemical Properties and Integrity (In Vitro)

**Figure 4:**
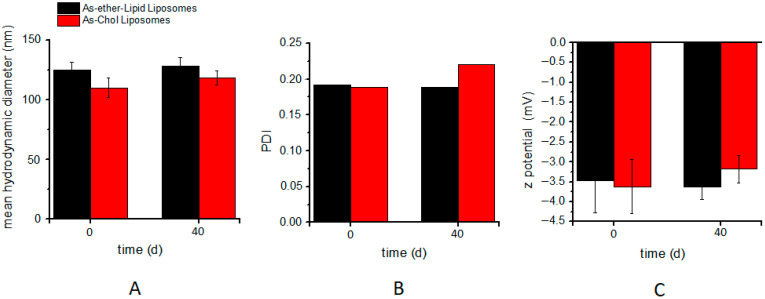
Vesicle mean hydrodynamic diameter (nm) (**A**), polydispersity Index (**B**) and ζ-potential (mV) (**C**) values of As-ether-lipid and As-Chol liposomes (they are the mean values from at least 5 measurements of 3 different preparations, in each case).

In order to test the potential of our novel arsonolipids as potential anticancer agents, we initially tested their ability to form arsonoliposomes, which are known to selectively kill cancer cells [28,33]. For the formation of novel arsonoliposomes, we used both types of the arsono synthesized compounds (As-ether-lipid **27** and As-Chol **32**), which were incorporated in the liposome bilayer of liposomes composed of DSPC, PEG2000, and Chol lipids. The molecular composition of the final liposomes that were used was: (a) DSPC/Chol/PEG2000/As-ether-lipid (38.7/31.9/5.2/24.2) (As-ether-lipid liposomes) and (b) DSPC/Chol/PEG2000/As-Chol (38.7/24.2/7.7/5.24/24.2) (As-Chol liposomes). Conventional arsonoliposomes with lipid composition DSPC/Chol/PEG2000/As-ester-lipid (38.7/31.9/5.2/24.2) (As-ester-lipid liposomes) were also prepared for comparison. The specific lipid composition was demonstrated previously to confer conventional arsonoliposomes with very high integrity [24]. The molar percentage of As-ether-lipid, As-Chol and As-ester-lipid in the different formulations was fixed to be the same. In both cases (As-ether-lipid liposomes and As-Chol liposomes), the liposomes that were formed were in the size range of nanoliposomes, with a mean hydrodynamic diameter of about 125 nm for As-lipid liposomes and 110 nm for As-Chol liposomes (Figure 4A). Polydispersity index values were around 0.19, indicating that highly uniform vesicles with respect to their particle size were formed (Figure 4B). The ζ-potential of both liposome types was about −3.47 mV for As-ether-lipid liposomes and −3.23 mV for As-Chol liposomes (Figure 4C), which is the expected value for such liposome preparations due to the fact that they are coated with PEG. Both liposome types were tested for their stability (size stability) during storage. The results showed that both liposome types were highly stable with respect to their mean diameter. In fact, both liposome types were found to retain their mean size after storage at 4 °C for up to 40 d (Figure 4A). Their polydispersity index and ζ-potential values were also stable (Figure 4B,C, respectively).

The results were found to be in line with previous conventional arsonoliposome preparations, which were found to have a mean hydrodynamic diameter of about 103.2 nm and ζ-potential of about −2.98 mV [24]. Similar values were recently measured for the same type of (conventional) asonoliposomes, with values of about 117.0 nm for their mean hydrodynamic diameter and PDI values of about 0.122. The corresponding ζ-potential was measured as about −3.55 mV [34].

**Figure 5:**
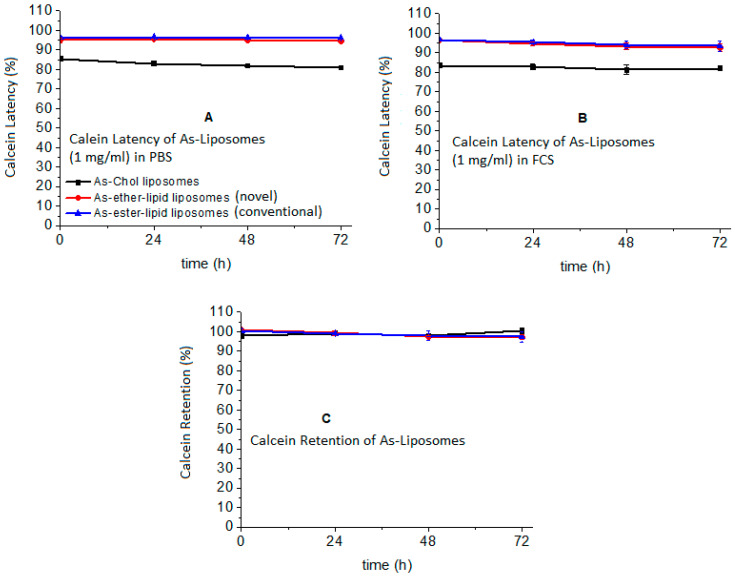
Calcein latency (%) in PBS (**A**) and FCS (**B**) as well as calcein retention (%) in vesicles containing As-ether-lipid **27** or As-Chol **32** (novel arsonoliposomes) during incubation at 37 °C (**C**). The previously synthesized As-ester-lipid liposomes of type **2** (conventional liposomes) were evaluated under identical conditions for comparison.

The arsono-containing liposomes that were formed (As-ether-lipid and As-Chol containing liposomes) were tested for their integrity during incubation in PBS 7.40 (control) as well as in 80% (*v/v*) Fetal Calf Serum (FCS). This information is important when considering liposomes for in vivo use as a measure to indicate the liposomes’ integrity in blood circulation. For this experiment, calcein (100 mM) was encapsulated in liposomes, and these were incubated at 37 °C in PBS and plasma proteins at a concentration of 1 mg lipid/mL of liposome dispersion (the lipid was measured by Stewart assay) [50]. The results are presented in Figure 5, where calcein latency (%) in PBS (**A**) and FCS (**B**) as well as calcein retention (%) (**C**) in the liposomes at various time points during the incubation period were calculated. It is evident that both types of arsono liposomes (As-ether-lipid liposomes and As-Chol liposomes) are highly intact, retaining their calcein content, for at least 72 h, which confirms their structural integrity and verifies that both liposome types could be considered for in vivo administration. Conventional arsonoliposomes (containing the As-Lipid of type **2** (As-ester-lipid)) were evaluated under identical conditions for comparison. The liposome composition was identical to those that have been used for As-ether-lipid liposomes and As-Chol liposomes (DSPC/Chol/PEG2000/As-ester-lipid (38.7/31.9/5.2/24.2)).

As seen from the liposome integrity results (Figure 5), the novel synthesized arsonoliposomes have similar -high- stability to the conventional arsonoliposomes for at least 72 h, both in buffer and serum proteins. The same applies for As-Chol liposomes, which were also found to be stable for at least 72 h. It should be mentioned that the lower latency values at time 0 that were noticed, both in buffer and serum, are probably due to looser attachment of calcein on the surface of this type of arsonoliposome or some calcein leakage from this type of liposome when they are diluted in PBS or FCS.

In summary, the results of the current studies prove that the novel synthesized arsonolipids are capable to form arsonoliposomes with diameters within the nano-range and narrow size distribution, absolutely comparable with conventional arsonoliposomes. Furthermore, the integrity of the novel arsonoliposome types tested herein during incubation in the absence and presence of serum proteins was demonstrated to be similar to that of conventional arsonoliposomes, ensuring their potential applicability as therapeutic nanosystems for in vivo use.

#### 3.2.2. Anticancer Potential of Novel Arsonoliposomes

A preliminary cytotoxicity study of the novel arsonoliposomes (As-ether-lipid **27** containing liposomes) against cancer cells was conducted, and their anticancer potential was evaluated in comparison with conventional arsonoliposomes (As-ester-lipid liposomes). In this study, two types of cancer cells were used in order to understand if the novel and conventional arsonoliposomes demonstrate similar (or not) cytotoxicity towards cancer cells with different glutathione concentrations since the mechanism of the anticancer activity of arsonoliposomes is related to their glutathione levels [24,37]. Although the exact glutathione concentrations of the two cell types used (PC3 and LLC cells) are not known, PC3 cells have been reported to have low glutathione levels [59], while lung cancer tumors were reported to have high levels [60].

The concentration of GSH is lower in normal tissue compared to tumors (2–20 μM in normal tissue and 0.5–10 mM in tumors) [61], and this has been linked with the selective cytotoxicity of arsonoliposomes towards cancer cells (compared to normal).

**Figure 6:**
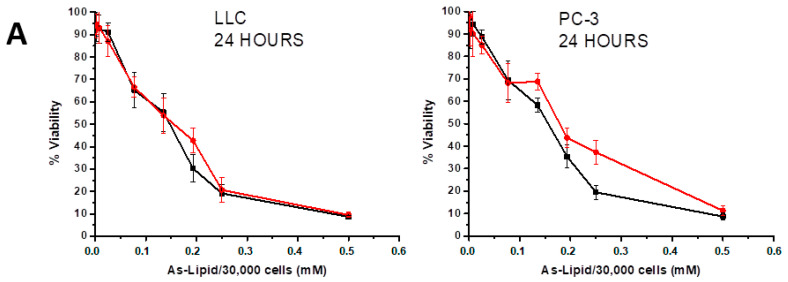

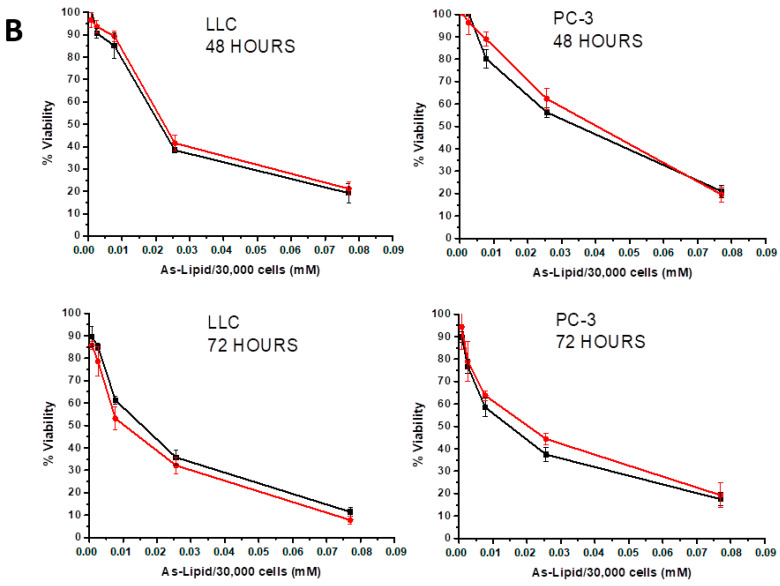
(**A**) Viability of LLC and PC-3 cancer cells after incubation with As-liposomes for 24 h at high concentrations (up to 0.5 mM As-Lipid/30,000 cells); (**Β**) viability of LLC and PC-3 cancer cells after incubation with As-liposomes for 48 and 72 h at low concentrations (up to 0.08 mM As-Lipid/30,000 cells). The black line refers to the As-ester-lipid liposomes of type **2**, while the red line refers to the As-ether-lipid liposomes of type **5**.

As described in detail in the Methods section, the As-ether-lipid liposomes were incubated with prostate cancer cells (PC-3) and Lewis lung carcinoma cells (LLC), and the reduction in cell viability after 24 h (for high arsonolipid concentrations) and after 48 and 72 h (for low arsonolipid concentrations) were measured by the MTT assay [53]. For comparison, the previously synthesized As-ester-lipid liposomes of type **2** (conventional arsonoliposomes) were studied under identical conditions. As seen (Figure 6), similar activity (which was dose- and time-dependent), was demonstrated between conventional and novel arsonoliposomes towards both types of cancer cells. Furthermore, the cytotoxicity of both arsonoliposome types was higher towards LLC cells compared to PC3 cells under all experimental conditions used (incubation period and arsonoliposomes concentrations), in good agreement with the information found about their glutathione levels [59,60,61]. Furthermore, in all cases, a clear dose-response of arsonoliposome cytotoxicity was observed, as demonstrated previously with other cell lines [27,28,31,32,34]. Furthermore, the current cytotoxicity results of conventional arsonoliposomes towards LLC cells are in good agreement with the ones acquired recently in the same cell line [34]. These results prove that the novel arsonolipid-containing arsonoliposomes retain the intrinsic anticancer activity of conventional arsonoliposomes, and thereby, the novel As-ether-lipid could be used as an alternative arsonolipid for the construction of arsonoliposomes. Whether or not the different structures of the novel arsonolipid may confer higher in vivo stability of the lipid and perhaps also of the arsonoliposomes containing them remain to be explored in in vivo studies.

## 4. Conclusions

The significance of this work can be summarized as follows: 

A new synthetic methodology is proposed, where 2-hydroxyethylarsonic acid is used as a key building block for an easy and efficient synthesis of arsono-containing compounds. Two novel types of arsonolipids were obtained, namely an ether type arsonolipid (As-ether-lipid) and an arsono cholesterol lipid (As-Chol lipid), and both types were found to form arsonoliposomes with high integrity, similar to that of conventional arsonoliposomes. 

Although the chemical hydrolytic stability of the As-ether-lipid (novel type arsonolipid) was not directly compared with that of the conventional arsonolipids (As-ester-lipid), it should be noted that the ether linkage between the alkyl groups and the glycerol group is expected to offer higher chemical hydrolytic stability as in the case of ether vs. ester phospholipids [42,43,44]. On the other hand, although a hydrolytic effect on the ester linkage between 2-hydroxyethylarsonic acid and the organic part of the novel arsonolipid is expected, the ease and the effect of such hydrolysis remains to be evaluated not only for the novel arsonolipids but also for the novel arsonoliposomes, mainly in in vivo studies. For now, the liposome integrity experiment has proven the high vesicle stability of the developed novel arsonoliposomes. Furthermore, the novel arsonoliposomes, composed by the ether type arsonolipid, showed similar toxicity towards two types of cancer cells to that of conventional arsonoliposomes, which were indicated to be glutathione level dependent, proving their intrinsic anticancer properties. 

The current results indicate that the new compounds may be used for the development of novel arsonoliposomes with at least similar integrity and anticancer activity compared to conventional arsonoliposomes. Whether or not the different structures of the novel arsonolipid compounds may confer higher in vivo stability of the lipids and perhaps also of the arsonoliposomes containing them remains to be explored by in vivo studies. In vivo studies should also be conducted to evaluate the safety of novel arsonoliposomes, although we do not expect that it will differ from that of conventional arsonoliposomes, for which no acute toxicity to the animals was realized (at the doses administered) in vivo [29,30,33].

## Supplementary Materials

The following supporting information can be downloaded at: https://www.mdpi.com/article/10.3390/pharmaceutics14081649/s1, NMR analysis of all compounds, as following: Figure S1: ^1^H NMR of octadecyl-1-methanesulfonate (**8**) in CDCl_3_; Figure S2: ^1^H NMR of *rac*-1-(2-(octadecyloxy)propoxy)octadecane (**10**) in CDCl_3_; Figure S3: ^1^H NMR of 2-hydroxyethylarsonic acid (**15**) in D_2_O; Figure S4: ^1^H NMR of diphenyl 2-hydroxyethylarsonodithionite (crude oily product) (**16**) in CDCl_3_; Figure S5: ^1^H NMR of 2-(palmitoyloxy)ethylarsonic acid (**18**) in CDCl_3_/CD_3_OD, 2:1; Figure S6: ^13^C NMR of 2-(palmitoyloxy)ethylarsonic acid (**18**) in CDCl_3_/CD_3_OD, 1:1; Figure S7: ^13^C NMR of 2-(palmitoyloxy)ethylarsonic acid (**18**) in CDCl_3_/DMSO-*d*_6_, 1:1; Figure S8: ^1^H NMR of 3-O-benzyl-sn-Glycerol / 3-(benzyloxy)propane-1,2-diol (**21**) in CDCl_3_; Figure S9: ^1^H NMR of *rac*-1-((2,3-bis(octadecyloxy)propoxy)methyl)benzene (**22**) in CDCl_3_; Figure S10: ^1^H NMR of *rac*-2,3-bis(octadecyloxy)propan-1-ol (**23**) in CDCl_3_; Figure S11: ^1^H NMR of *rac*-2,3-bis(octadecyloxy)propanoic acid (**24**) in CDCl_3_; Figure S12: ^1^H NMR of *rac*-2-(2,3-bis(octadecyloxy)ethylarsonic acid [As-Lipid] (**27**) in CDCl_3_; Figure S13: ^13^C NMR of *rac*-2-(2,3-bis(octadecyloxy)ethylarsonic acid [As-Lipid] (**27**) in CDCl_3_; Figure S14: ^1^H-^13^C HSQC of *rac*-2-(2,3-bis(octadecyloxy)ethylarsonic acid [As-Lipid] (**27**) in CDCl_3_; Figure S15. ^1^H NMR of *rac*-2,3-bis(octadecyloxy)propanoic acid (**24**) vs. ^1^H NMR of *rac*-2-(2,3-bis(octadecyloxy)ethylarsonic acid [As-Lipid] (**27**) in CDCl_3_, as analyzed in Figure S11 and Figure S12; Figure S16: ^1^H NMR of cholesterol-3-O-succinic acid monoester (**29**) in CDCl_3_; Figure S17: ^13^C NMR of cholesterol-3-O-succinic acid monoester (**29**) in CDCl_3_; Figure S18: ^1^H NMR of As-Chol (**32**) in CDCl_3_; Figure S19: ^13^C NMR of As-Chol (**32**) in CDCl_3_; Figure S20: ^1^H NMR of Cholesterol-3-O-succinic acid monoester (**29**) vs. ^1^H NMR of As-Chol (**32**) in CDCl_3_, as analyzed in Figures S16 and S18; ESI-MS analysis of compounds: **8**, **10**, **18**, **22**, **23**, **24**, **27**, **29**, **32**.
