# Supplementary material for: Synthesis of Novel Arsonolipids and Development of Novel Arsonoliposome Types
Source: Pharmaceutics. 2022 Aug 8;14(8):1649. doi: 10.3390/pharmaceutics14081649 (PMC9416600; doi:10.3390/pharmaceutics14081649)
Supplement: Supplementary file 1 [file pharmaceutics-14-01649-s001.zip › pharmaceutics-1820508-supplementary.pdf]

pharmaceutics

MDPI

Supplementary Materials: Synthesis of Novel Arsonolipids and Development of Novel Arsonoliposome Types

Spyridon Mourtas, Konstantina Papadia, Golfo G. Kordopati, Panayiotis V. Ioannou, Sophia G. Antimisiaris and Gerasimos M. Tsivgoulis

Octadecyl-1-methanesulfonate (8)

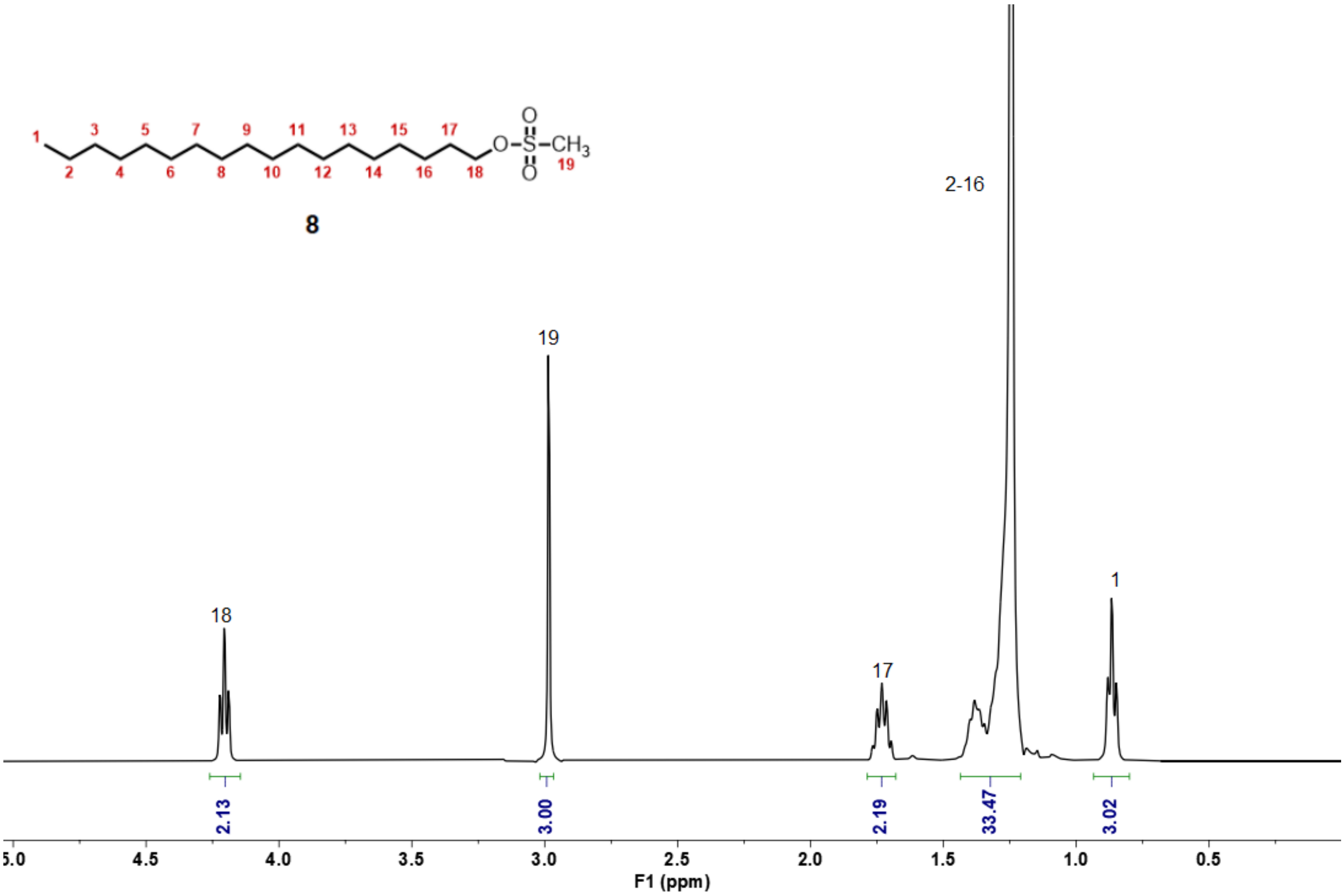

Figure S1. ^{1}H NMR of octadecyl-1-methanesulfonate (**8**) in $CDCl_3$.

^{1}H NMR (400 MHz, $CDCl_3$) δ (ppm): 4.21 (t, $^3J_{H17H18}$ = 6.6 Hz, 2H; H_{18}), 2.99 (s, 3H; H_{19}), 1.73 (tt, $^3J_{H16H17}$ = 6.6 Hz, $^3J_{H17H18}$ = 6.6 Hz, 2H; H_{17}), 1.40-1.20 (br, 30H; H_2-H_{16}), 0.87 (t, $^3J_{H1H2}$ = 6.7 Hz, 3H; H_1).

ESI-MS (m/z) calcd for $C_{19}H_{40}NaO_3S$ [M+Na]$^+$: 371.26; found: 371.93.

rac-1-(2-(octadecyloxy) propoxy)octadecane (10)

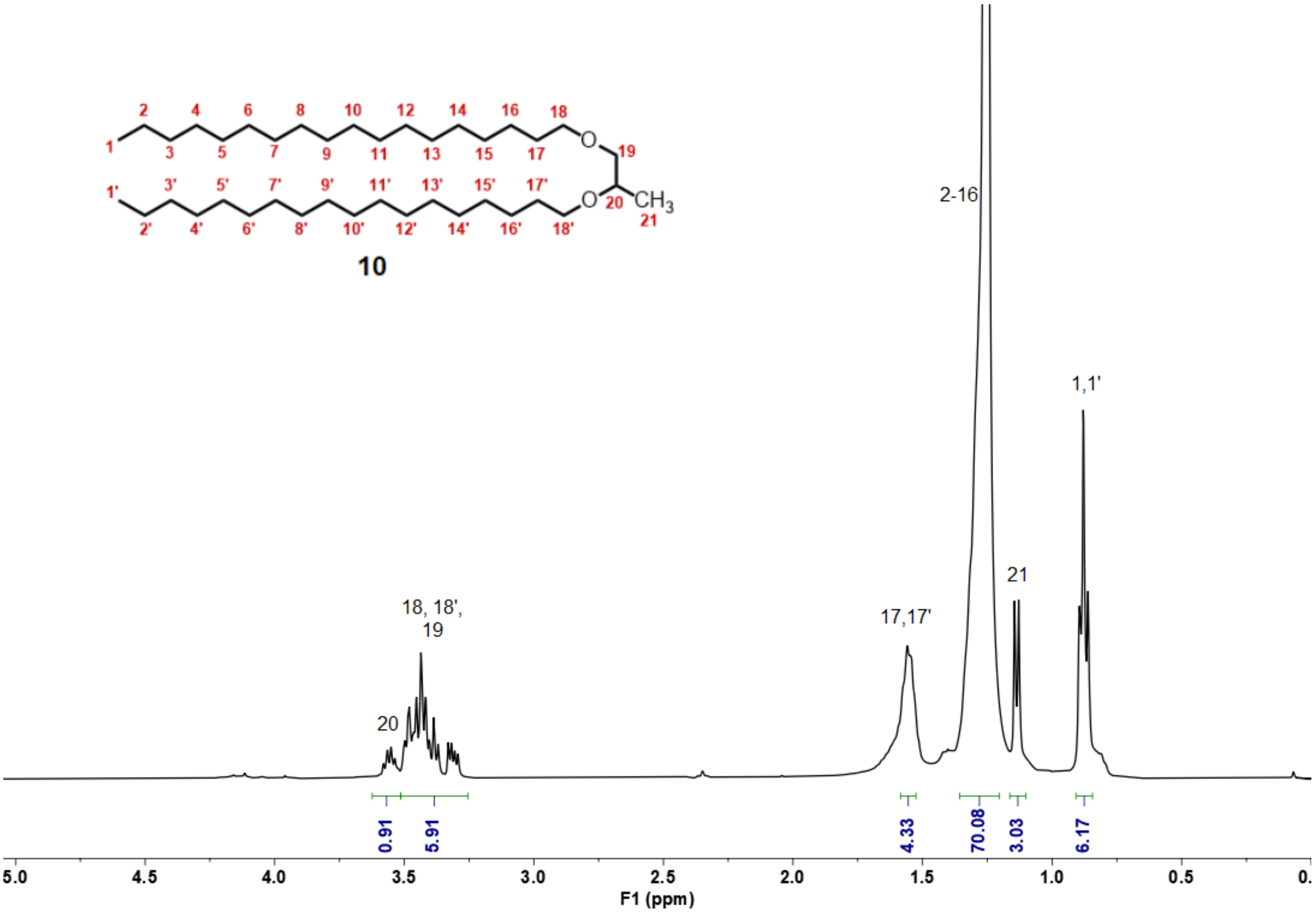

Figure S2. ^{1}H NMR of *rac*-1-(2-(octadecyloxy)propoxy)octadecane (**10**) in $CDCl_3$.

^{1}H NMR (400 MHz, $CDCl_3$) δ (ppm): 3.58-3.51 (m, 1H; H_{20}), 3.51-3.29 (m, 6H; H_{18}, $H_{18'}$, H_{19}), 1.61-1.51 (m, 4H; H_{17}, $H_{17'}$), 1.60-1.20 (br, 60H overlapped with H_2O; H_2-H_{16}, $H_{2'}$-$H_{16'}$), 1.14 (d, $^3J_{H20H21}$ = 6.3 Hz, 3H; H_{21}), 0.88 (t, $^3J_{H1H2}$=$^3J_{H1'H2'}$= 6.7 Hz, 6H; H_1, $H_{1'}$).

ESI-MS (m/z): calcd for $C_{39}H_{81}O_2$ $[M+H]^+$: 581.62, found: 581.80, calcd for $C_{39}H_{80}O_2Na$ $[M+Na]^+$: 603.61, found: 603.78, calcd for $C_{39}H_{80}KO_2$ $[M+K]^+$: 619.58, found: 619.84.

2-hydroxyethylarsonic acid (15)

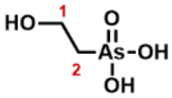

15

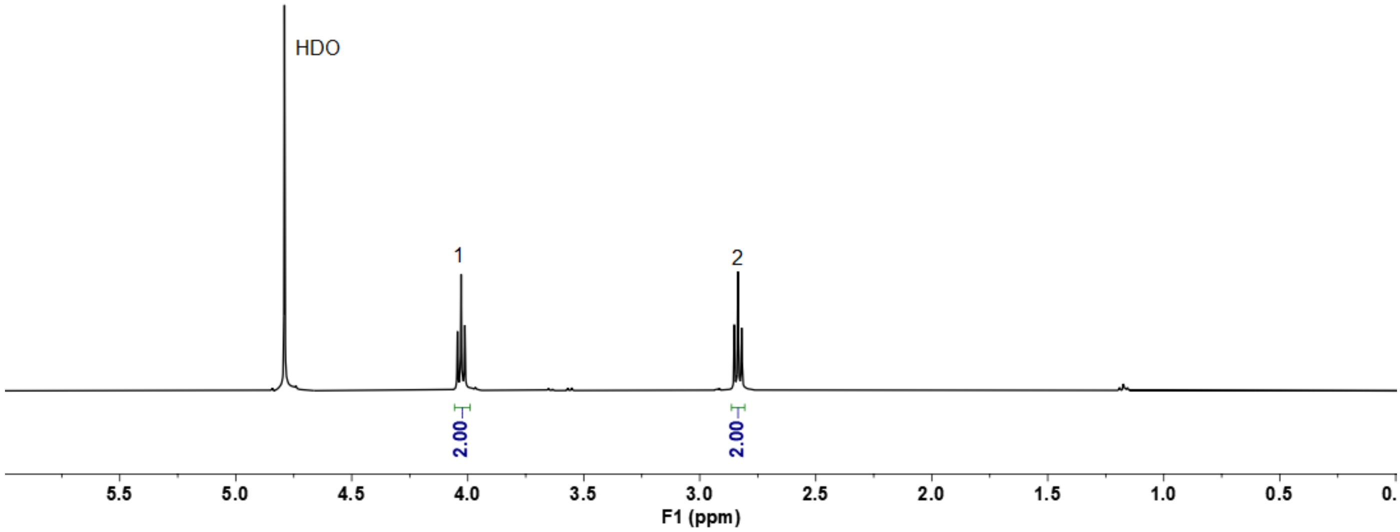

Figure S3. ^1H NMR of 2-hydroxyethylarsonic acid (**15**) in D_2O.

^1H NMR (400 MHz, D_2O) δ (ppm): 4.03 (t, $^3J_{H1H2}$ = 6.4 Hz, 2H; H_1), 2.84 (t, $^3J_{H1H2}$ = 6.4 Hz, 2H; H_2).

diphenyl 2-hydroxyethylarsonodithionite (16)

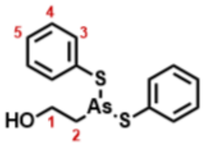

16

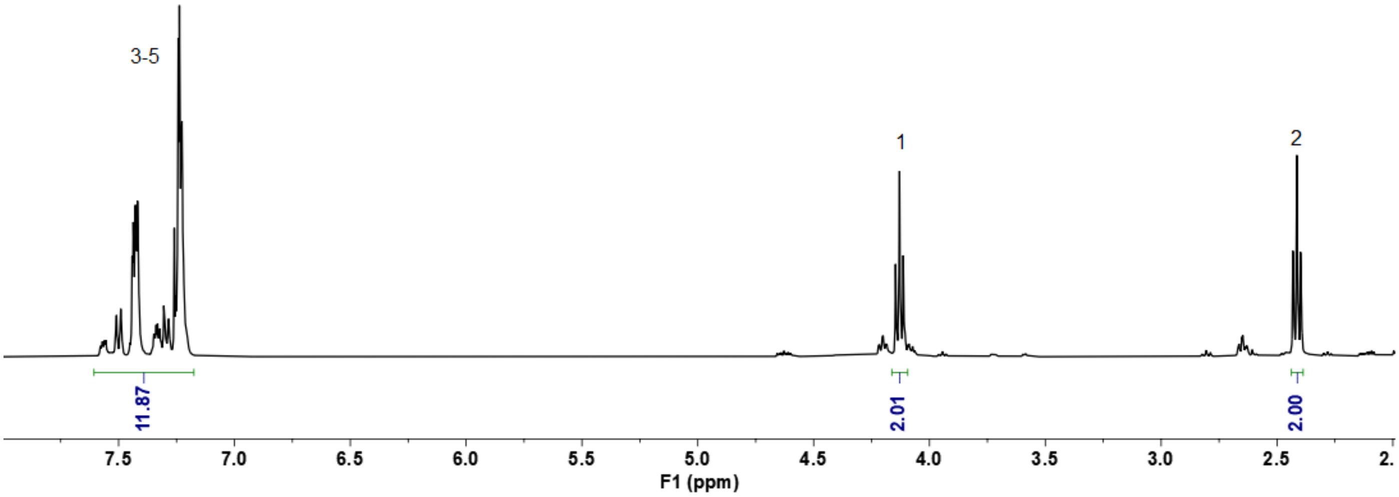

Figure S4. ^{1}H NMR of diphenyl 2-hydroxyethylarsonodithionite (crude oily product) (**16**) in $CDCl_3$.

^{1}H NMR (400 MHz, $CDCl_3$, crude oily product) δ (ppm): 7.61-7.18 (m, 10H overlapped with $CHCl_3$; H_3-H_5), 4.13 (t, $^{3}J_{H1H2}$ = 6.6 Hz, 2H; H_1), 2.41 (t, $^{3}J_{H1H2}$ = 6.6 Hz, 2H; H_2).

2-(palmitoyloxy)ethylarsonic acid (18)

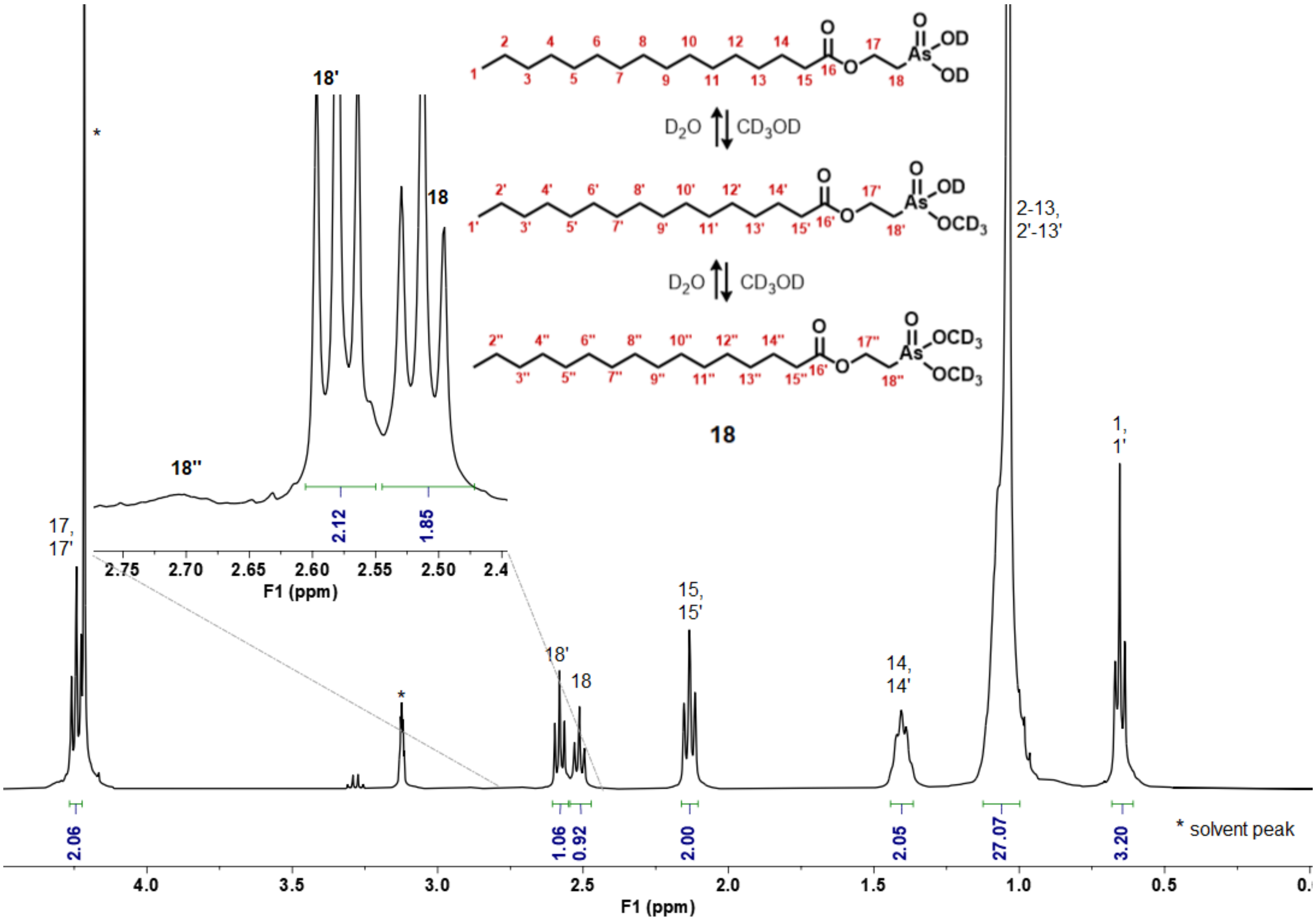

Figure S5. ^{1}H NMR of 2-(palmitoyloxy)ethylarsonic acid (**18**) in $CDCl_3/CD_3OD$, 2:1.

^{1}H NMR (400 MHz, $CDCl_3/CD_3OD$ 2:1) δ (ppm): 4.24 (t, $^3J_{H17H18}$ = 6.6 Hz, $^3J_{H17'H18'}$ = 6.6 Hz, 2H; H_{17}, $H_{17'}$), [2.58 (t, $^3J_{H17'H18'}$ = 6.6 Hz; $H_{18'}$), 2.51 (t, $^3J_{H17H18}$ = 6.6 Hz; H_{18}), 2H], 2.13 (t, $^3J_{H14H15}$ = 6.6 Hz, $^3J_{H14'H15'}$ = 6.6 Hz, 2H; H_{15}, $H_{15'}$), 1.44-1.67 (m, 2H; H_{14}, $H_{14'}$), 1.13-1.00 (bs, 24H overlapped with -OH, H_2-H_{13}, $H_{2'}$-$H_{13'}$), 0.65 (t, $^3J_{H1H2}$ = 6.7 Hz, $^3J_{H1'H2'}$ = 6.7 Hz, 3H; H_1, $H_{1'}$).

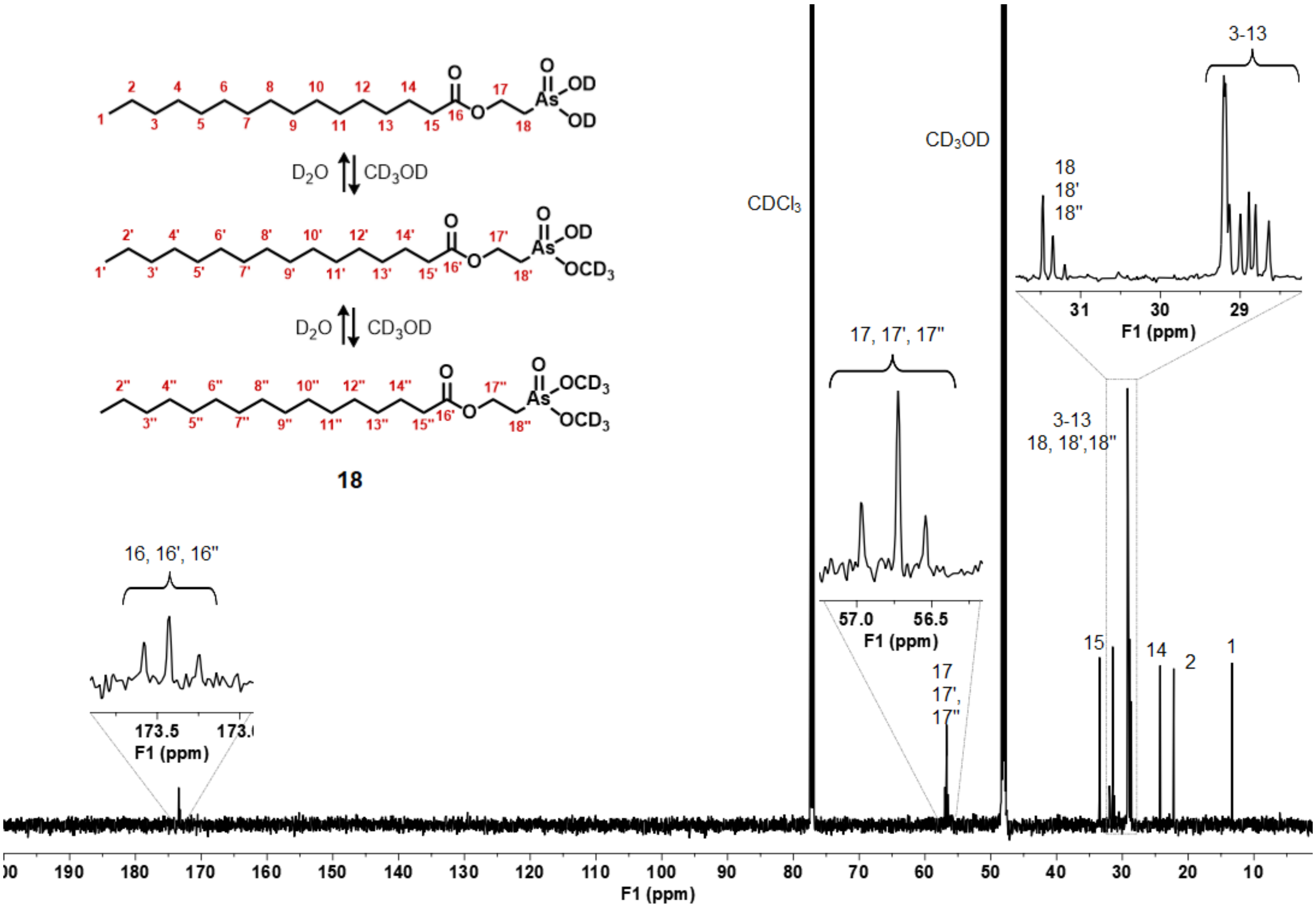

Figure S6. ^{13}C NMR of 2-(palmitoyloxy)ethylarsonic acid (**18**) in $CDCl_3/CD_3OD$, 1:1.

^{13}C NMR (151 MHz, $CDCl_3/CD_3OD$ 1:1) δ (ppm): [173.6, 173.4, 173.2 (C_{16}, $C_{16'}$, $C_{16''}$)], [57.0, 56.7, 56.5, (C_{17}, $C_{17'}$, $C_{17''}$)], 33.5 (C_{15}), [31.5, 31.3, 31.2 (C_{18}, $C_{18'}$, $C_{18''}$)], [29.2, 29.2, 29.1, 29.0, 28.9, 28.8, 28.6 (C_3-C_{13})], 24.2 (C_{14}), 22.2 (C_2), 13.3 (C_1).

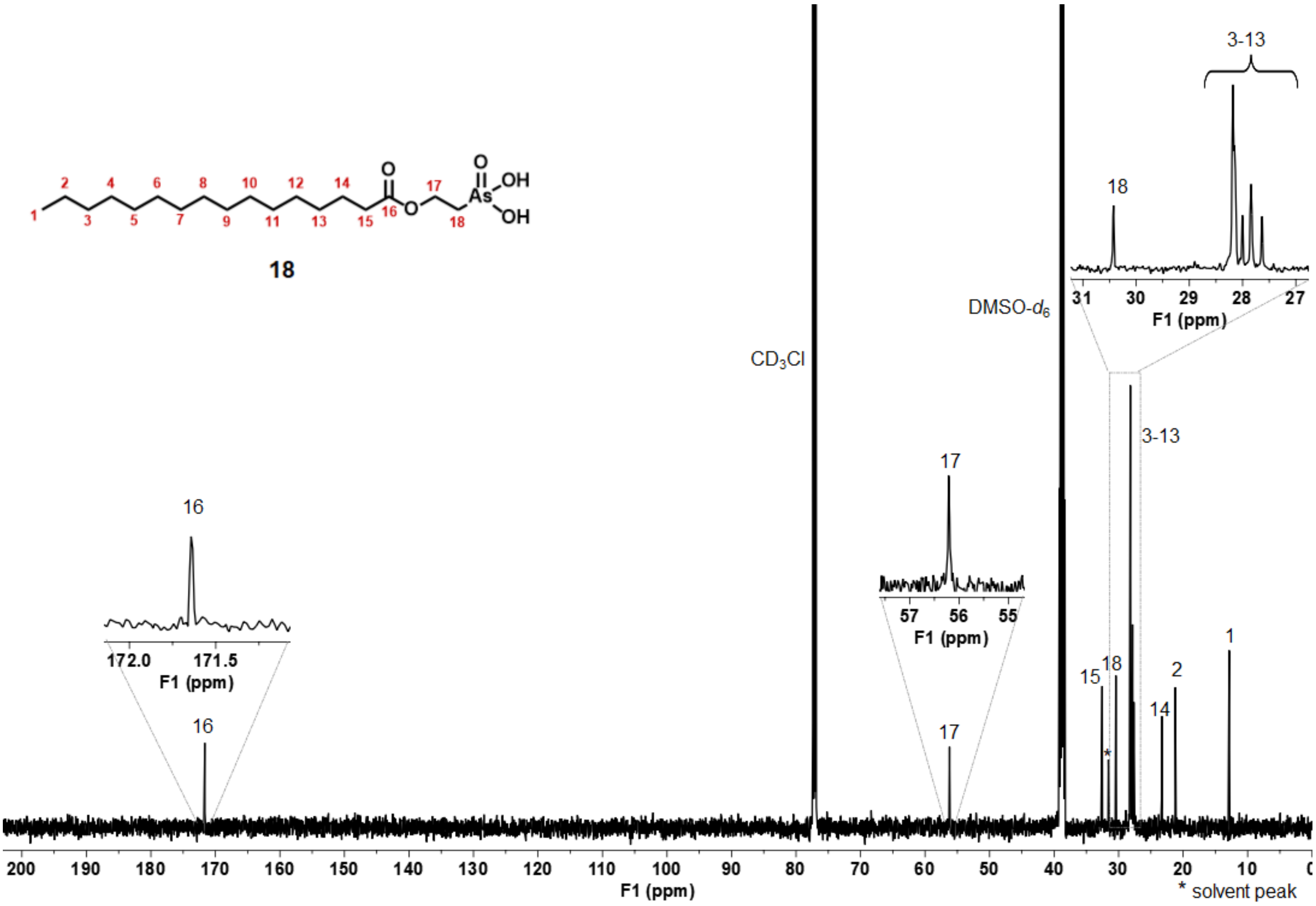

Figure S7. ^{13}C NMR of 2-(palmitoyloxy)ethylarsonic acid (**18**) in $CDCl_3$/DMSO-d_6, 1:1.

^{13}C NMR (151 MHz, $CDCl_3$/ DMSO-d_6 1:1) δ (ppm): 171.6 (C_{16}), 56.2 (C_{17}), 32.5 (C_{15}), 31.6 (C_{18}), [28.2, 28.0, 27.8, 27.6 (C_3-C_{13})], 23.3 (C_{14}), 21.2 (C_2), 12.9 (C_1).

ESI-MS (m/z): calcd for $C_{19}H_{40}AsO_5$ [M(OH,OCH_3)+H]$^+$: 423.21, found: 423.75, calcd for $C_{20}H_{41}AsO_5$ [M(OCH_3,OCH_3)+H]$^+$: 437.22, found: 437.72, calcd for $C_{20}H_{41}AsNaO_5$ [(OCH_3,OCH_3)+Na]$^+$: 459.21, found: 459.74, calcd for $C_{20}H_{41}AsKO_5$ [M(OCH_3,OCH_3)+K]$^+$: 475.18, found: 475.58, calcd for $C_{40}H_{82}As_2NaO_{10}$ [2M(OCH_3,OCH_3)+Na]$^+$: 895.42, found: 895.63.

3-O-benzyl-sn-Glycerol / 3-(benzyloxy)propane-1,2-diol (21)

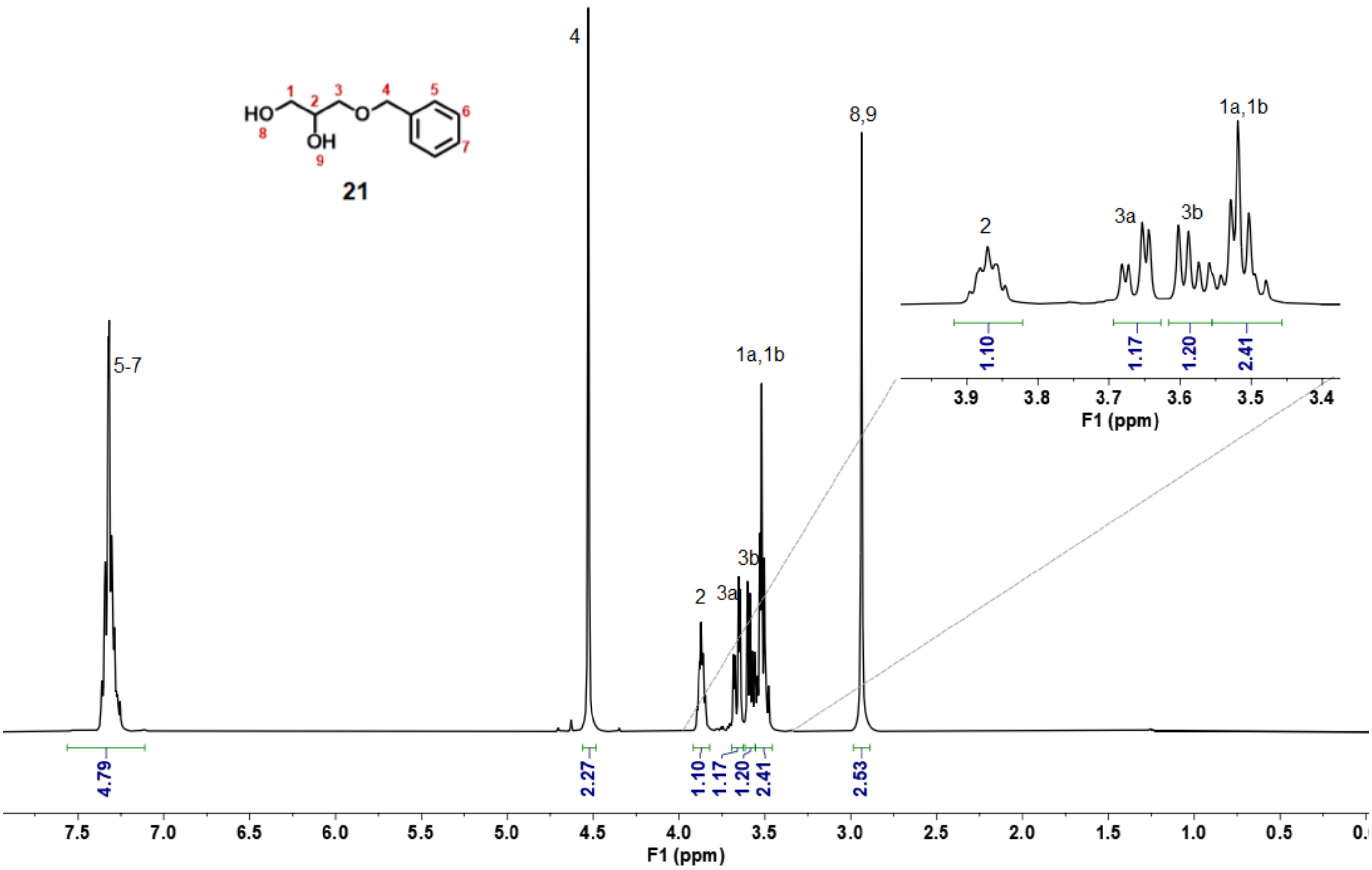

Figure S8. ^{1}H NMR of 3-O-benzyl-sn-Glycerol / 3-(benzyloxy)propane-1,2-diol (**21**) in $CDCl_3$.

^{1}H NMR (400 MHz, $CDCl_3$) δ (ppm): 7.48-7.12 (m, 5H; H_5-H_7), 4.53 (s, 2H; H_4), 3.90-3.82 (X part of of an ABXMN system, 1H; H_2), 3.66 (A part of an ABXMN system, $^3J_{H3aH2}$ = 3.7 Hz, $^2J_{H3aH3b}$ = -11.5 Hz, 1H; H_{3a}), 3.58 (B part of an ABXMN system, $^2J_{H3bH2}$ = 5.8 Hz, $^3J_{H3aH3b}$ = -11.5 Hz, 1H; H_{3b}), 3.53 (M part of an ABXMN system, $^3J_{H1aH2}$ = 4.0 Hz, $^2J_{H1aH1b}$ = -9.7 Hz, 1H; H_{1a}), 3.50 (N part of an ABXMN system, $^3J_{H1bH2}$ = 6.5 Hz, $^2J_{H1aH1b}$ = -9.7 Hz, 1H; H_{1b}), 2.94 (s, 2H; H_8, H_9).

rac-1-((2,3-bis(octadecyloxy)propoxy)methyl)benzene (22)

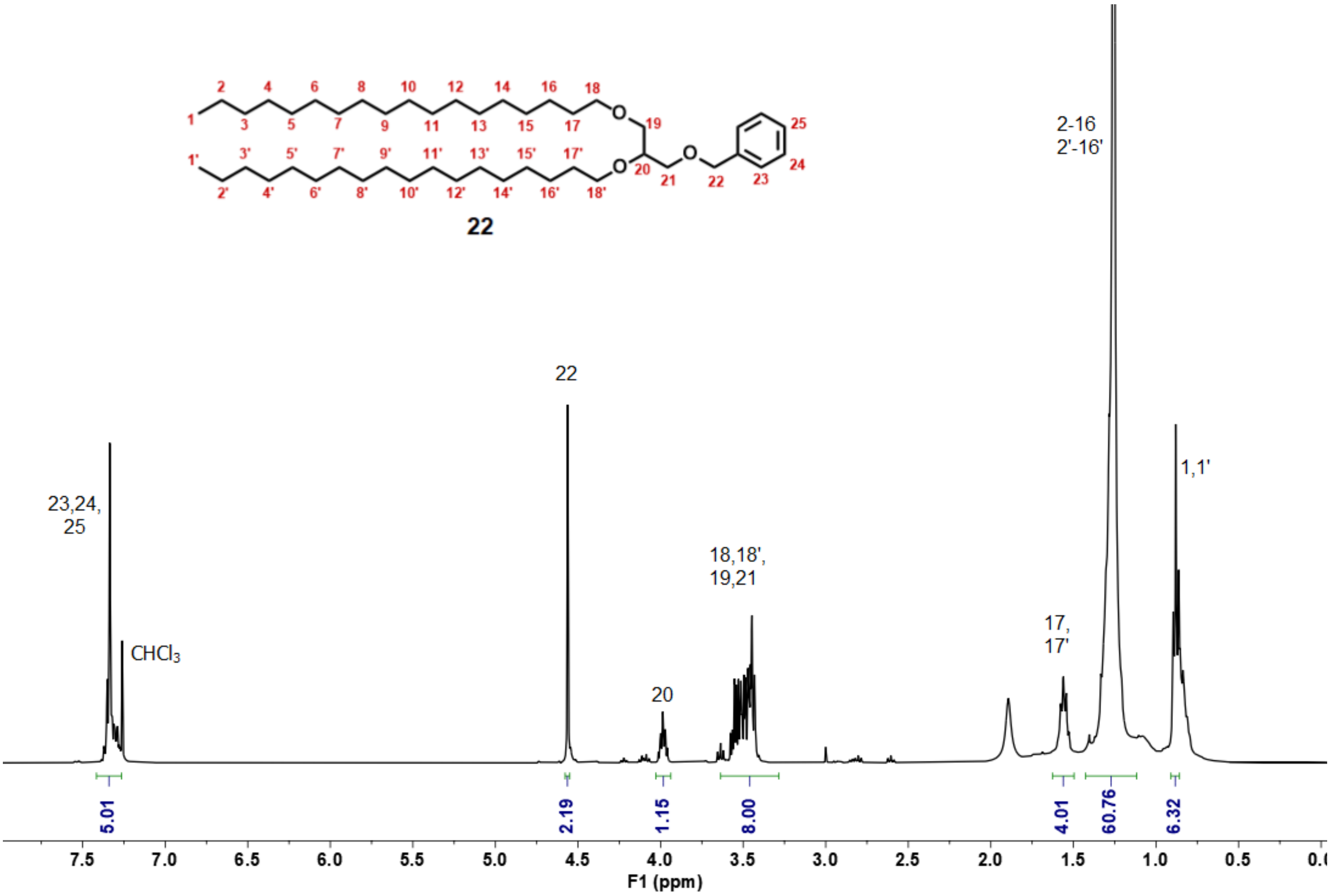

Figure S9. ^{1}H NMR of *rac*-1-((2,3-bis(octadecyloxy)propoxy)methyl)benzene (**22**) in $CDCl_3$.

^{1}H NMR (400 MHz, $CDCl_3$) δ (ppm): 7.41-7.27 (m, 5H; H_{23}-H_{25}), 4.56 (s, 2H; H_{22}), 4.01-3.96 (m, 1H; H_{20}), 3.65-3.42 (m, 8H; H_{18}, $H_{18'}$, H_{19}, H_{21}), 1.63-1.50 (m, 4H; H_{17}, $H_{17'}$), 1.43-1.12 (br, 60H; H_2-H_{16}, $H_{2'}$-$H_{16'}$), 0.88 (t, $^3J_{H1H2}$ = 6.7 Hz, 6H; H_1, $H_{1'}$).

ESI-MS (m/z): calcd for $C_{46}H_{87}O_3$ $[M+H]^+$: 687.6655, found: 687.98, calcd for $C_{46}H_{86}NaO_3$ $[M+Na]^+$: 709.6475, found: 710.00, calcd for $C_{46}H_{86}O_3K$ $[M+K]^+$: 725.62, found: 725.98.

rac-2,3-bis(octadecyloxy)propan-1-ol (23)

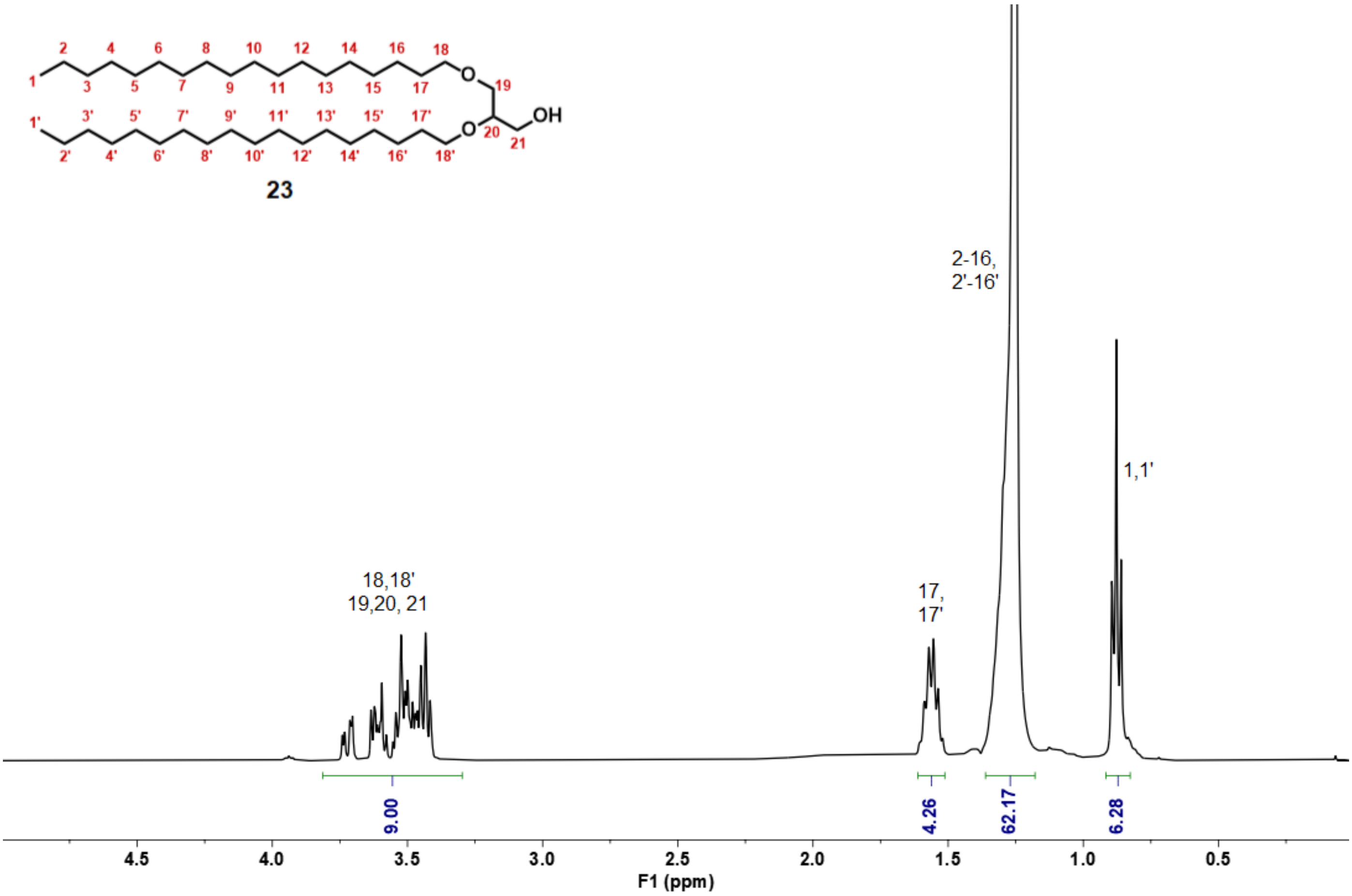

Figure S10. ^{1}H NMR of *rac*-2,3-bis(octadecyloxy)propan-1-ol (**23**) in $CDCl_3$.

^{1}H NMR (400 MHz, $CDCl_3$) δ (ppm): 3.72-3.42 (m, 9H; H_{18}, $H_{18'}$, H_{19}, H_{20}, H_{21}), 1.60-1.52 (m, 4H; H_{17}, $H_{17'}$), 1.33-1.25 (br, 60H; H_2-H_{16}, $H_{2'}$-$H_{16'}$), 0.88 (t, $^3J_{H1H2}$ = 6.7 Hz, 6H; H_1, $H_{1'}$).

ESI-MS (m/z): calcd for $C_{39}H_{80}NaO_3$ $[M+Na]^+$: 619.60, found: 619.89.

***rac*-2,3-bis(octadecyloxy)propanoic acid (24)**

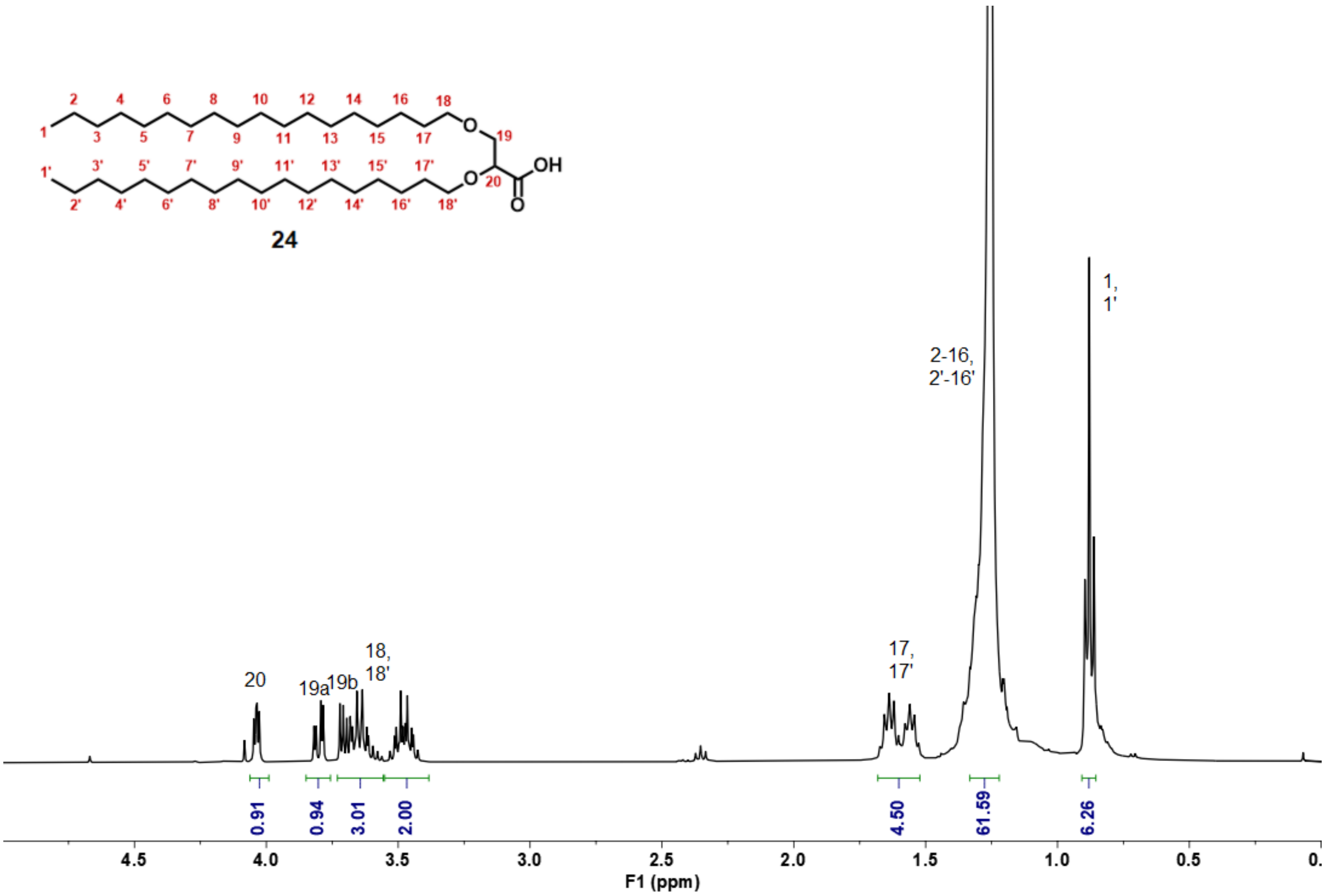

Figure S11. ^{1}H NMR of *rac*-2,3-bis(octadecyloxy)propanoic acid (**24**) in $CDCl_3$.

^{1}H NMR (400 MHz, $CDCl_3$) δ (ppm): 4.04 (X part of an ABX system, $^3J_{H19aH20}$ = 5.1 Hz, $^3J_{H19bH20}$ = 3.1 Hz, 1H; H_{20}), 3.80 (A part of an ABX system, $^3J_{H19aH20}$ = 3.1 Hz, $^2J_{H19aH19b}$ = -10.5 Hz, 1H; H_{19a}), 3.7 (B part of an ABX system, $^3J_{H19bH20}$ = 5.1 Hz, $^2J_{H19aH19b}$ = -10.5 Hz, 1H; H_{19b}, overlapped with other signals), 3.75-3.37 (m, 4H; H_{18}, $H_{18'}$, overlapped with the signal of H_{19b}), 1.67-1.53 (m, 4H; H_{17}, $H_{17'}$), 1.33-1.22 (br, 60H; H_2-H_{16}, $H_{2'}$-$H_{16'}$), 0.88 (t, $^3J_{H1H2}$ = 6.7 Hz, 6H; H_1, $H_{1'}$).

ESI-MS (m/z) calcd for $C_{39}H_{78}NaO_4$ $[M+Na]^+$: 633.58, found: 633.63, calcd for $C_{39}H_{78}O_4K$ $[M+K]^+$: 649.55, found: 649.60, calcd for $C_{78}H_{156}NaO_8$ $[2M+Na]^+$: 1244.17, found: 1244.46, calcd for $C_{78}H_{156}O_8K$ $[2M+K]^+$: 1260.14, found: 1260.32.

***rac*-2-(2,3-bis(octadecyloxy)ethylarsonic acid [As-Lipid] (27)**

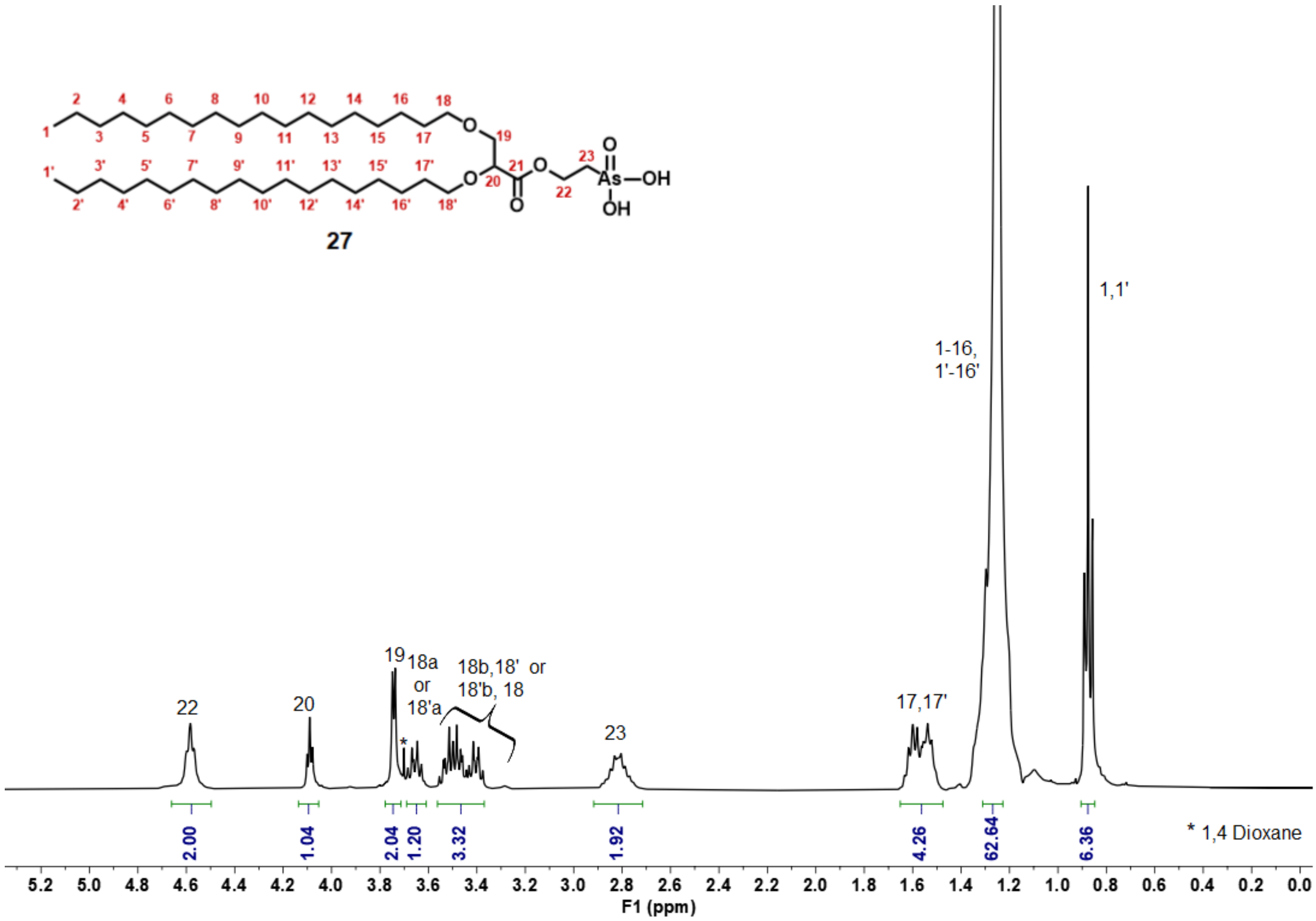

Figure S12. ^{1}H NMR of *rac*-2-(2,3-bis(octadecyloxy)ethylarsonic acid [As-Lipid] (**27**) in $CDCl_3$.

^{1}H NMR (400 MHz, $CDCl_3$) δ (ppm): 4.58 (t, $^3J_{H22H23}$ = 7.0 Hz, 2H; H_{22}), 4.09 (t, $^3J_{H19H20}$ = 4.6 Hz, 1H; H_{20}), 3.74 (d, $^3J_{H19H20}$ = 4.6 Hz, 2H; H_{19}), 3.69-3.61 (m, 1H; H_{18a} or $H_{18'a}$), 3.56-3.37 [m, 3H; (H_{18b}, $H_{18'}$ or $H_{18'b}$, H_{18})], 2.92-2.72 (m, 2H; H_{23}), 1.65-1.48 (m, 4H; H_{17}, $H_{17'}$), 1.31-1.23 (br, 60H; H2; H_2-H_{16}, $H_{2'}$-$H_{16'}$), 0.87 (t, $^3J_{H1H2}$ = 6.7 Hz, 6H; H_1, $H_{1'}$).

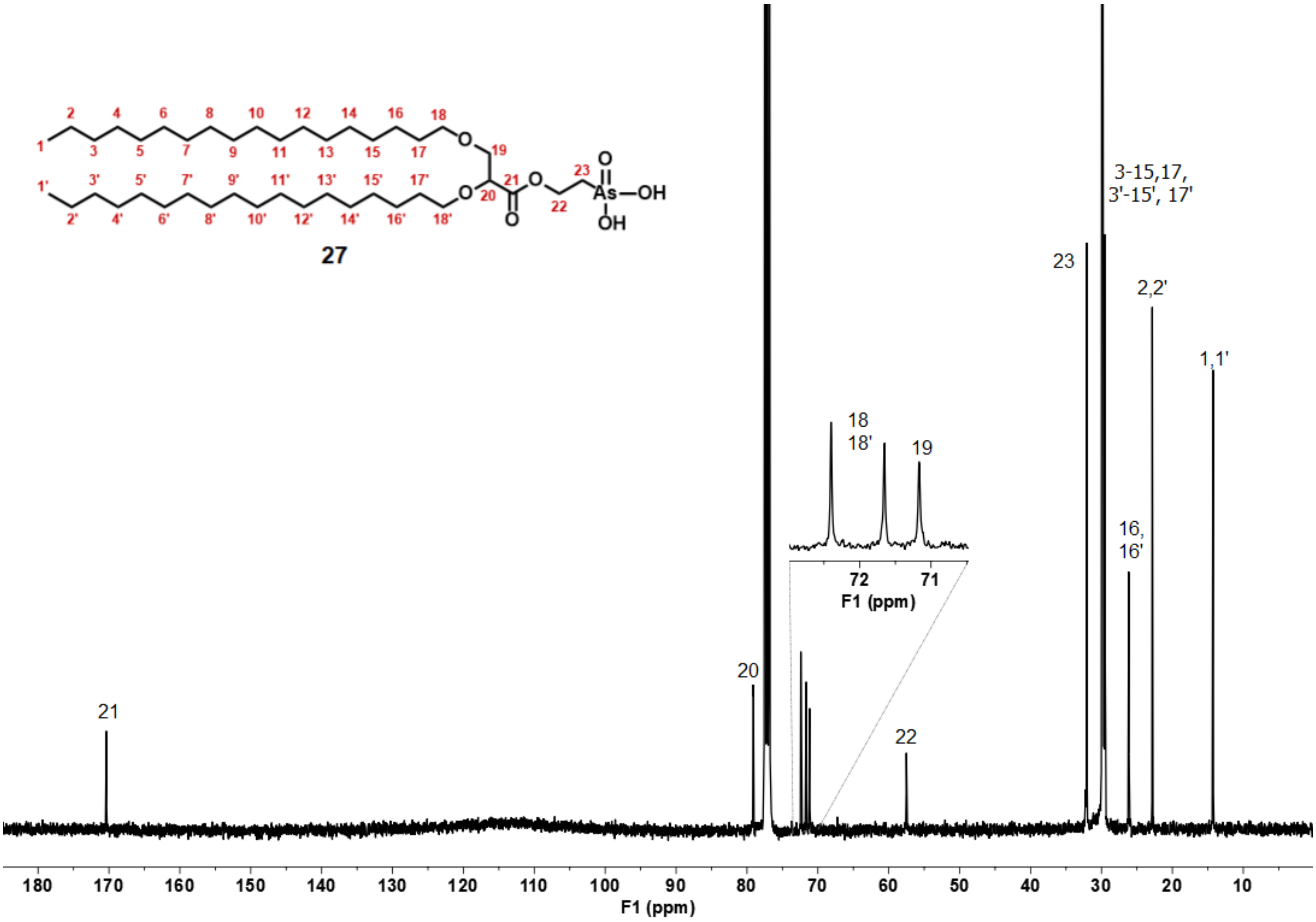

Figure S13. ^{13}C NMR of *rac*-2-(2,3-bis(octadecyloxy)ethylarsonic acid [As-Lipid] (**27**) in $CDCl_3$.

^{13}C NMR (101 MHz, $CDCl_3$) δ (ppm): 170.4 (C_{21}), 79.1 (C_{20}), [72.4, 71.7 (C_{18}, $C_{18'}$), 71.2 (C_{19}), 57.5 (C_{22}), 32.1(C_{23}), [29.9, 29.8, 29.7, 29.6, 29.6, 29.5; (C_3-C_{15}, C_{17}, $C_{3'}$-$C_{15'}$)], [26.2, 26.1; (C_{16} ,$C_{16'}$)], 22.8 (C_2 ,$C_{2'}$), 14.3 (C_1 ,$C_{1'}$).

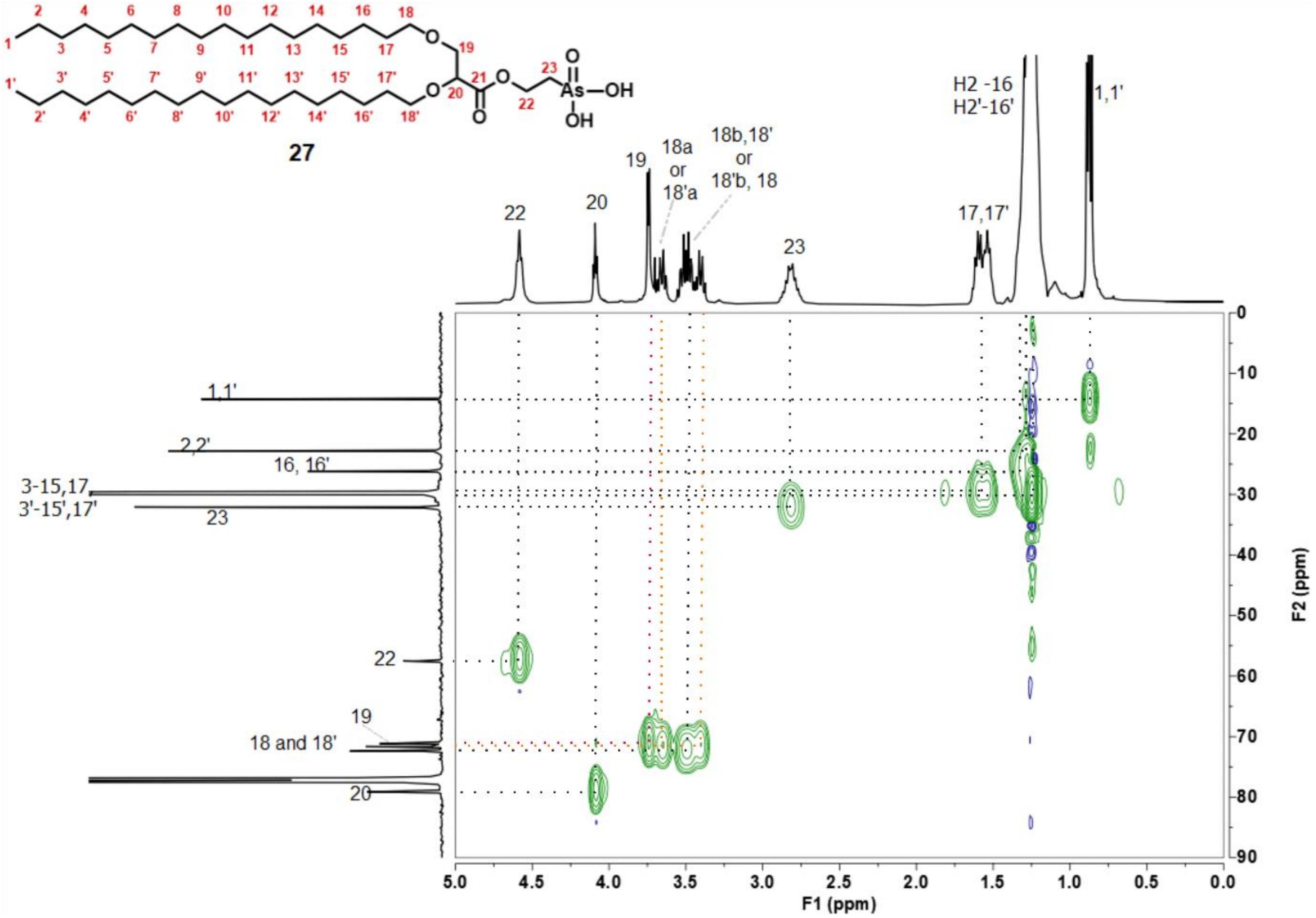

Figure S14. ^{1}H-^{13}C HSQC of *rac*-2-(2,3-bis(octadecyloxy)ethylarsonic acid [As-Lipid] (**27**) in $CDCl_3$.

^{1}H NMR (400 MHz, $CDCl_3$) δ (ppm): 4.58 (t, $^{3}J_{H22H23}$ = 7.0 Hz, 2H; H_{22}), 4.09 (t, $^{3}J_{H19H20}$ = 4.6 Hz, 1H; H_{20}), 3.74 (d, $^{3}J_{H19H20}$ = 4.6 Hz, 2H; H_{19}), 3.69-3.61 (m, 1H; H_{18a} or $H_{18'a}$), 3.56-3.37 [m, 3H; (H_{18b}, $H_{18'}$ or $H_{18'b}$, H_{18})], 2.92-2.72 (m, 2H; H_{23}), 1.65-1.48 (m, 4H; H_{17}, $H_{17'}$), 1.31-1.23 (br, 60H; H2; H_2-H_{16}, $H_{2'}$-$H_{16'}$), 0.87 (t, $^{3}J_{H1H2}$ = 6.7 Hz, 6H; H_1, $H_{1'}$).

^{13}C NMR (101 MHz, $CDCl_3$) δ (ppm): 170.4 (C_{21}), 79.1 (C_{20}), [72.4, 71.7 (C_{18}, $C_{18'}$), 71.2 (C_{19}), 57.5 (C_{22}), 32.1(C_{23}), [29.9, 29.8, 29.7, 29.6, 29.6, 29.5; (C_3-C_{15}, C_{17}, $C_{3'}$-$C_{15'}$)], [26.2, 26.1; (C_{16} ,$C_{16'}$)], 22.8 (C_2 ,$C_{2'}$), 14.3 (C_1 ,$C_{1'}$).

ESI-MS (m/z) calcd for $C_{42}H_{86}AsO_7$ $[M(OH,OCH_3)+H]^+$: 777.56, found: 799.88, calcd for $C_{42}H_{85}AsNaO_7$ $[M(OH,OCH_3)+Na]^+$: 799.54, found: 799.832, calcd for $C_{43}H_{88}AsO_7$ $[M(OCH_3,OCH_3)+H]^+$: 791.57, found: 791.84, calcd for $C_{43}H_{87}AsNaO_7$ $[M(OCH_3,OCH_3)+Na]^+$: 813.56, found: 813.87, calcd for $C_{43}H_{87}AsKO_7$ $[M(OCH_3,OCH_3)+K]^+$: 829.53, found: 829.85, calcd for $C_{86}H_{174}As_2KO_{14}$ $[2M(OCH_3,OCH_3)+K]^+$: 1604.10, found: 1604.75.

rac-2,3-bis(octadecyloxy)propanoic acid (24) vs rac-2-(2,3-bis(octadecyloxy)ethylarso-nic acid [As-Lipid] (27)

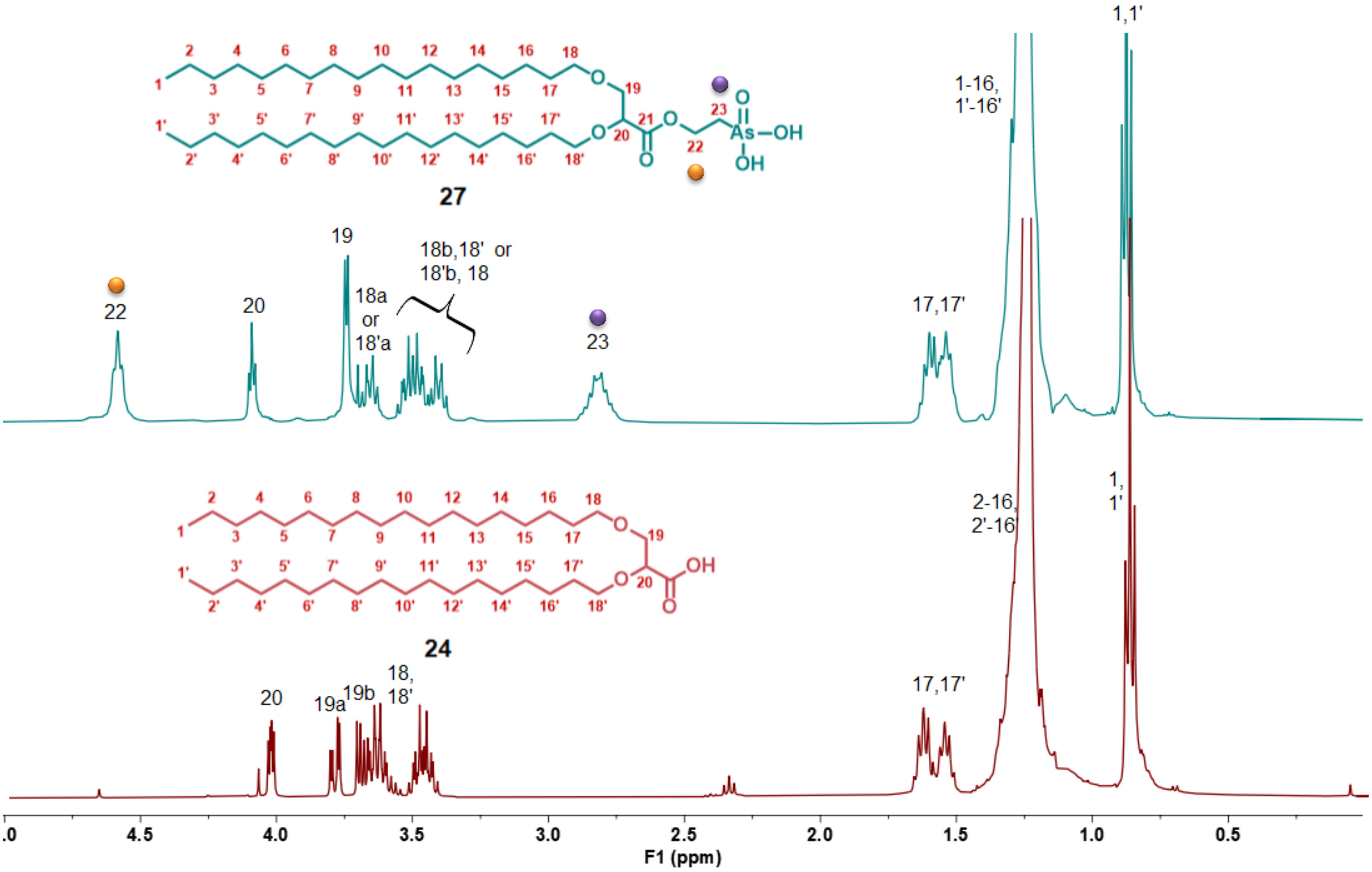

Figure S15. ^{1}H NMR of *rac*-2,3-bis(octadecyloxy)propanoic acid (**24**) vs ^{1}H NMR of *rac*-2-(2,3-bis(octadecyloxy)ethylarsonic acid [As-Lipid] (**27**) in $CDCl_3$, as analyzed in Figure S11 and Figure S12.

Cholesterol-3-O-succinic acid monoester (29)

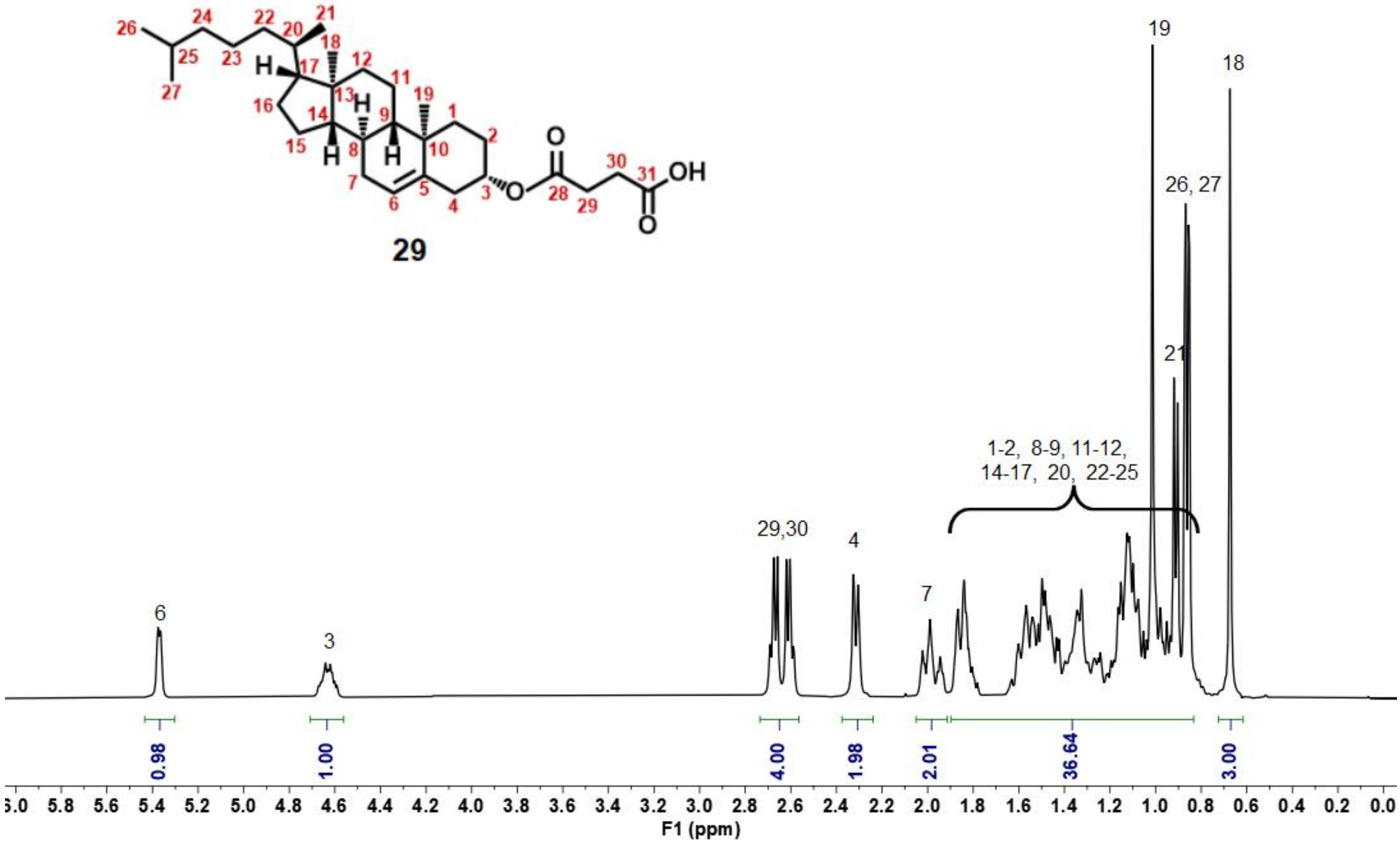

Figure S16. ^{1}H NMR of cholesterol-3-O-succinic acid monoester (**29**) in $CDCl_3$.

^{1}H NMR (400 MHz, $CDCl_3$) δ (ppm): 5.37 (d, $^3J_{H6H7a}$ = 5.0 Hz, $^3J_{H6H7b}$ = 0 Hz, 1H; H_6), 4.68-4.55 (m, 1H; H_3), 2.72-2.56 (m, 4H; H_{29}, H_{30}), 2.31 (d, $^3J_{H3H4}$ = 8.1 Hz, 2H; H_4), 2.05-1.92 (m, 2H; H_7), 1.90-0.85 (overlapping signals, 36H; $H_{1\text{-}2}$, $H_{8\text{-}9}$, $H_{11\text{-}12}$, $H_{14\text{-}17}$, $H_{19\text{-}27}$), 1.01 (s, 3H; H_{19} overlapped with other signals), 0.91 (d, $^3J_{H20H21}$ = 6.5 Hz 3H; H_{21} overlapped with other signals), 0.88-0.85 (two doublets, $^3J_{H25H26}$ = $^3J_{H25H27}$ = 6.7 Hz, 6H; $H_{26\text{-}27}$), 0.67 (s, 3H; H_{18}).

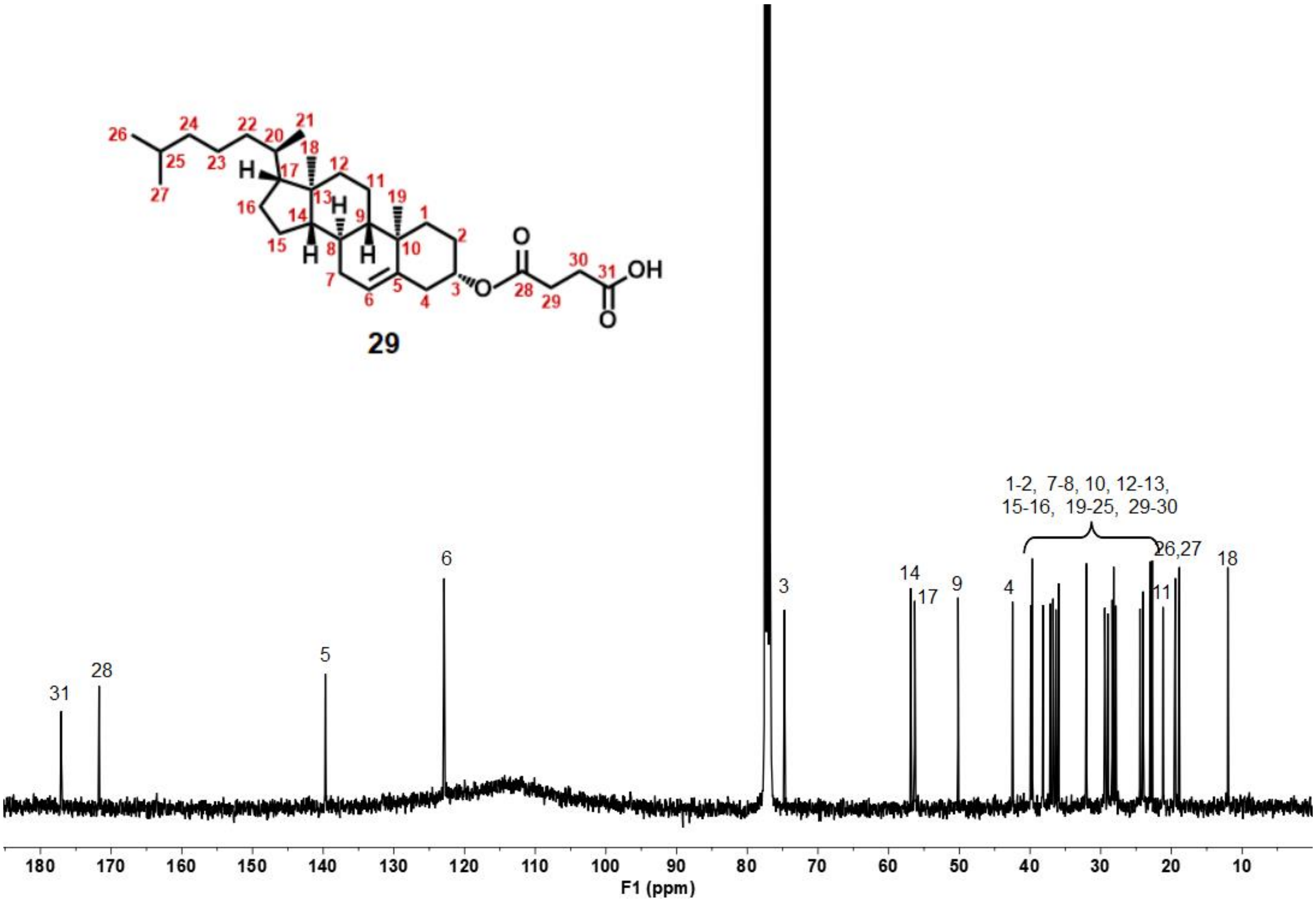

Figure S17. ^{13}C NMR of cholesterol-3-O-succinic acid monoester (**29**) in $CDCl_3$.

^{13}C NMR (101 MHz, $CDCl_3$) δ (ppm): 177.1 (C_{31}), 171.7 (C_{28}), 139.7 (C_5), 122.9 (C_6), 74.7 (C_3), 56.9(C_{14}), 56.3 (C_{17}), 50.2 (C_9), 42.5 (C_4), [39.9, 39.7, 38.2, 37.1, 36.7, 36.4, 36.0, 32.1, 32.0, 29.4, 29.0, 28.4, 28.2, 27.9, 24.4, 24.0, 23.0, 22.7, 21.2 (C_{1-2}, C_{7-8}, C_{10}, C_{12-13}, C_{15-16}, C_{19-25}, C_{29-30})], [19.5 and 18.9 (C_{26} and C_{27})], 12.0 (C_{18}).

ESI-MS (m/z) calcd for $C_{31}H_{50}NaO_4$ $[M+Na]^+$: 509.36, found: 509.91, calcd for $C_{31}H_{50}O_4K$ $[M+K]^+$: 525.33, found: 525.82, calcd for $C_{62}H_{100}NaO_8$ $[2M+Na]^+$: 995.73, found: 996.11.

As-Chol (32)

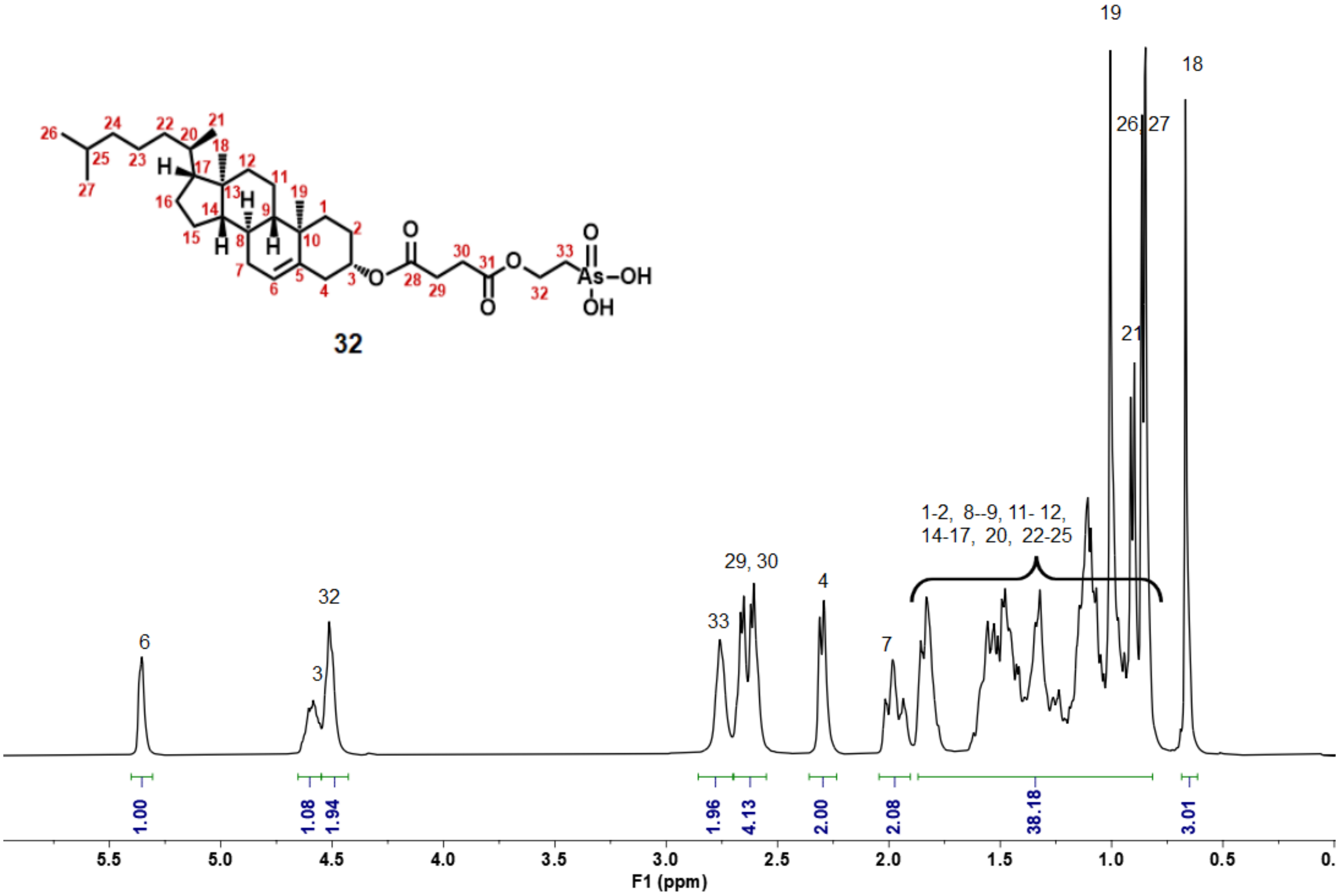

Figure S18. ^{1}H NMR of As-Chol (**32**) in $CDCl_3$.

^{1}H NMR (400 MHz, $CDCl_3$) δ (ppm): 5.36 (d, $^{3}J_{H6H7a}$ = 5.1 Hz, $^{3}J_{H6H7b}$ = 0 Hz, 1H; H_6), 4.67-4.55 (m, 1H; H_3), 4.55-4.40 (t, $^{3}J_{H32H33}$ = 6.7 Hz, 2H; H_{32}), 2.83-2.70 (m, $^{3}J_{H32H33}$ = 6.7 Hz, 2H; H_{33}), 2.70-2.54 (m, 4H; H_{29}, H_{30}), 2.30 (d, $^{3}J_{H3H4}$ = 8.0 Hz, 2H; H_4), 2.05-1.90 (m, 2H; H_7), 1.89-0.81 (overlapping signals, 36H; H_{1-2}, H_{8-9}, H_{11-12}, H_{14-17}, H_{19-27}), 1.01 (s, 3H; H_{19} overlapped with other signals), 0.91 (d, $^{3}J_{H20H21}$ = 6.4 Hz 3H; H_{21} overlapped with other signals), 0.88-0.82 (two doublets, $^{3}J_{H25H26}$ = $^{3}J_{H25H27}$ = 6.5 Hz, 6H; $H_{26,}$ H_{27}), 0.67 (s, 3H; H_{18}).

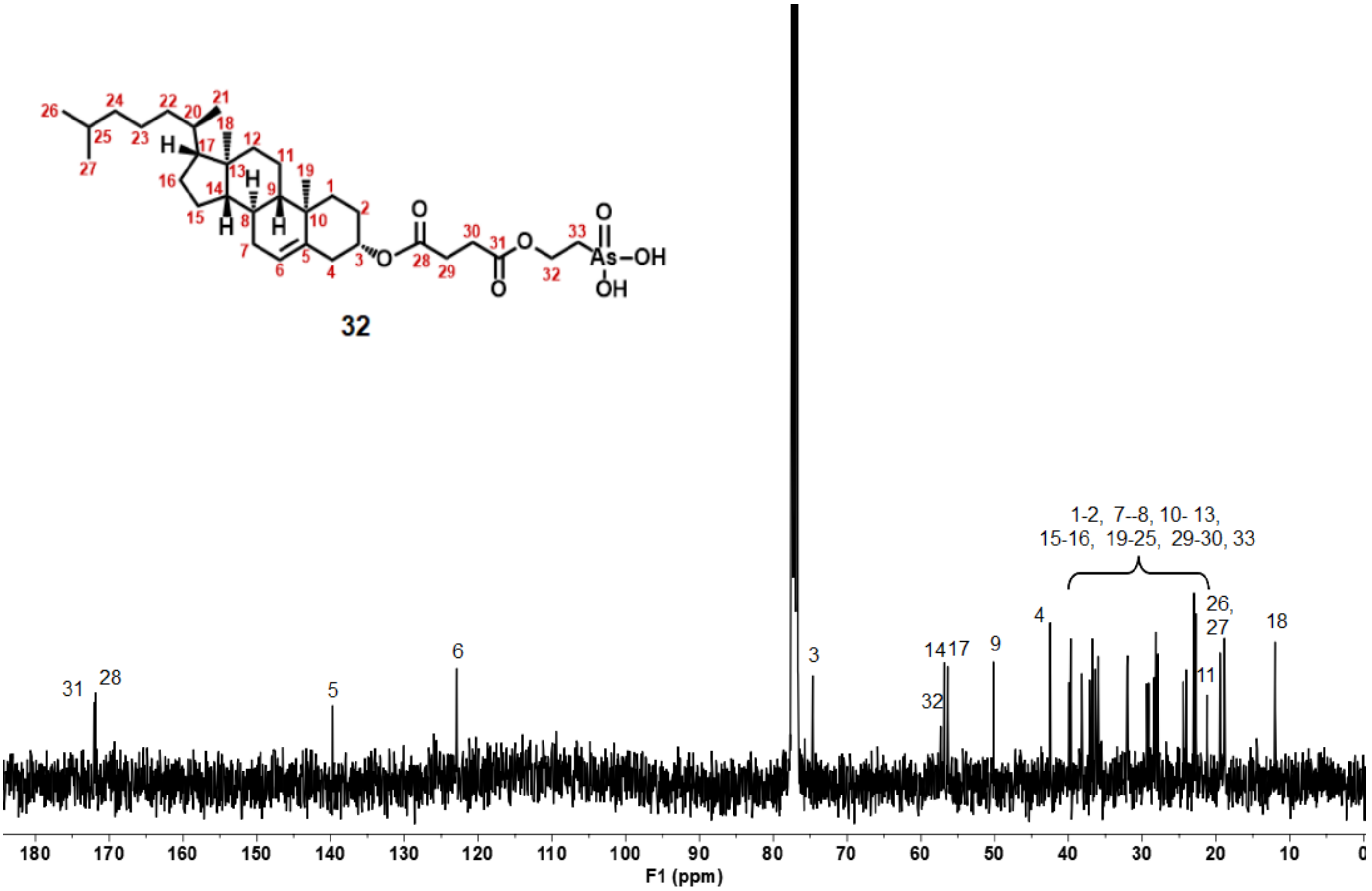

Figure S19. ^{13}C NMR of As-Chol (**32**) in $CDCl_3$.

^{13}C NMR (101 MHz, $CDCl_3$) δ (ppm): 172.1 (C_{31}), 171.8 (C_{28}), 139.7 (C_5), 122.9 (C_6), 74.6 (C_3), 57.3 (C_{32}), 56.8(C_{14}), 56.3 (C_{17}), 50.2 (C_9), 42.5 (C_4), [39.9, 39.7, 38.2, 37.1, 36.7, 36.4, 36.0, 32.1, 32.0, 29.4, 29.1, 28.4, 28.2, 27.9, 24.4, 24.0, 23.0, 22.7, 21.2 (C_{1-2}, C_{7-8}, C_{10-13}, C_{15-16}, C_{19-25}, C_{29-30}, C_{33})], [19.5 and 18.9 (C_{26} and C_{27})], 12.0 (C_{18}).

ESI-MS (m/z) calcd for $C_{34}H_{57}AsNaO_7$ $[M(OH,OCH_3)+Na]^+$: 675.32, found: 675.46, calcd for $C_{35}H_{60}AsO_7$ $[M(OCH_3,OCH_3)+H]^+$: 667.36, found: 667.47, calcd for $C_{35}H_{59}AsNaO_7$ $[M(OCH_3,OCH_3)+Na]^+$: 689.34, found: 689.49, calcd for $C_{35}H_{59}AsKO_7$ $[M(OCH_3,OCH_3)+K]^+$: 705.31, found: 705.69, calcd for $C_{70}H_{118}As_2NaO_{14}$ $[2M(OCH_3,OCH_3)+Na]^+$: 1355.69, found: 1355.71.

Cholesterol-3-O-Succinic Acid monoester (29) vs As-Chol (32)

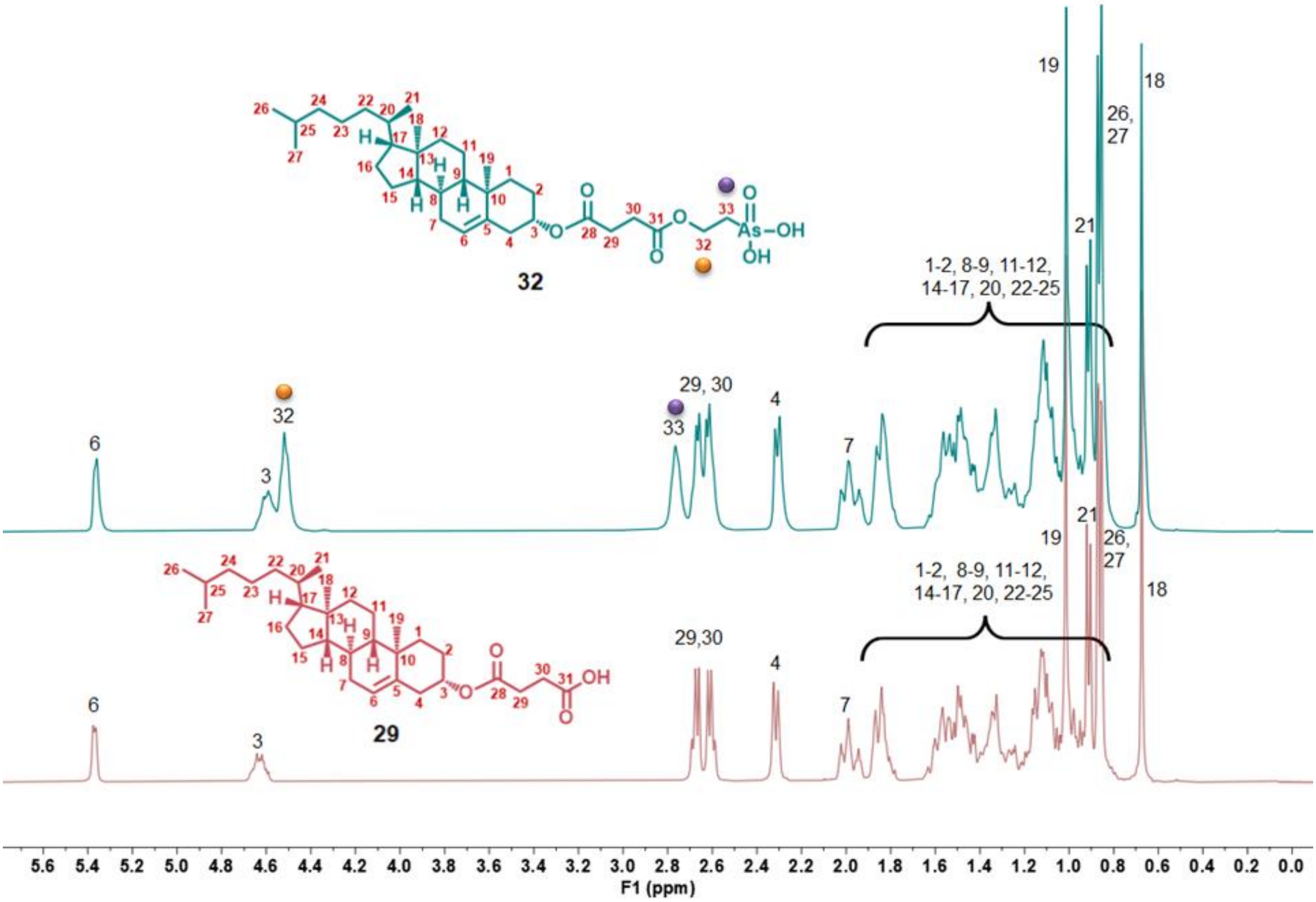

Figure S20. ^{1}H NMR of Cholesterol-3-O-succinic acid monoester (**29**) vs ^{1}H NMR of As-Chol (**32**) in $CDCl_3$, as analyzed in Figure S16 and Figure S18.
